# Genomic surveillance of SARS-CoV-2 evolution by a centralised pipeline and weekly focused sequencing, Austria, January 2021 to March 2023

**DOI:** 10.2807/1560-7917.ES.2024.29.23.2300542

**Published:** 2024-06-06

**Authors:** Olga Frank, David Acitores Balboa, Maria Novatchkova, Ezgi Özkan, Marcus Martin Strobl, Ramesh Yelagandula, Tanino Guiseppe Albanese, Lukas Endler, Fabian Amman, Vera Felsenstein, Milanka Gavrilovic, Melanie Acosta, Timothej Patocka, Alexander Vogt, Ido Tamir, Julia Klikovits, Alexander Zoufaly, Tamara Seitz, Manuela Födinger, Andreas Bergthaler, Alexander Indra, Daniela Schmid, Peter Klimek, Alexander Stark, Franz Allerberger, Bernhard Benka, Katharina Reich, Luisa Cochella, Ulrich Elling

**Affiliations:** 1 Institute of Molecular Biotechnology of the Austrian Academy of Science (IMBA), Vienna BioCenter (VBC), Vienna, Austria; 2 Research Institute of Molecular Pathology (IMP), Vienna BioCenter (VBC), Vienna, Austria; 3 Max Perutz Laboratories, University of Vienna, Department of Biochemistry and Cell Biology, Vienna BioCenter (VBC), Vienna, Austria; 4 CeMM Research Center for Molecular Medicine of the Austrian Academy of Science, Vienna, Austria; 5 Institute of Hygiene and Applied Immunology, Department of Pathophysiology, Infectiology and Immunology, Medical University of Vienna, Vienna, Austria; 6 Vienna Biocenter Core Facilities GmbH (VBCF), Vienna, Austria; 7 Österreichische Agentur für Gesundheit und Ernährungssicherheit (AGES), Vienna, Austria; 8 Department of Medicine, Klink Favoriten, Vienna, Austria; 9 Sigmund Freud Private University, Vienna, Austria; 10 Institute of Laboratory Diagnostics, Clinic Favoriten, Vienna, Austria; 11 Department of infection diagnostics and infectious disease epidemiology, Medical University of Vienna, Austria; 12 Complexity Science Hub Vienna, Vienna, Austria Section for Science of Complex Systems, CeMSIIS, Medical University of Vienna, Vienna, Austria; 13 Medical University of Vienna, Vienna BioCenter (VBC), Vienna, Austria; 14 Federal Ministry of Social Affairs, Health, Care and Consumer Protection, Vienna; 15 Present address: Department of Molecular Biology and Genetics, Johns Hopkins University School of Medicine, Baltimore, MD, United States

**Keywords:** SARS-CoV-2, Covid-19, pandemic preparedness, disease X, genomic surveillance, nowcasting, SARSeq, Austria, NGS

## Abstract

**Background:**

The COVID-19 pandemic was largely driven by genetic mutations of SARS-CoV-2, leading in some instances to enhanced infectiousness of the virus or its capacity to evade the host immune system. To closely monitor SARS-CoV-2 evolution and resulting variants at genomic-level, an innovative pipeline termed SARSeq was developed in Austria.

**Aim:**

We discuss technical aspects of the SARSeq pipeline, describe its performance and present noteworthy results it enabled during the pandemic in Austria.

**Methods:**

The SARSeq pipeline was set up as a collaboration between private and public clinical diagnostic laboratories, a public health agency, and an academic institution. Representative SARS-CoV-2 positive specimens from each of the nine Austrian provinces were obtained from SARS-CoV-2 testing laboratories and processed centrally in an academic setting for S-gene sequencing and analysis.

**Results:**

SARS-CoV-2 sequences from up to 2,880 cases weekly resulted in 222,784 characterised case samples in January 2021–March 2023. Consequently, Austria delivered the fourth densest genomic surveillance worldwide in a very resource-efficient manner. While most SARS-CoV-2 variants during the study showed comparable kinetic behaviour in all of Austria, some, like Beta, had a more focused spread. This highlighted multifaceted aspects of local population-level acquired immunity. The nationwide surveillance system enabled reliable nowcasting. Measured early growth kinetics of variants were predictive of later incidence peaks.

**Conclusion:**

With low automation, labour, and cost requirements, SARSeq is adaptable to monitor other pathogens and advantageous even for resource-limited countries. This multiplexed genomic surveillance system has potential as a rapid response tool for future emerging threats.

**Key public health message**

**What did you want to address in this study and why?**

During the COVID-19 pandemic, mutations in the genome of SARS-CoV-2, the virus causing COVID-19, resulted in some instances in more infectious SARS-CoV-2 variants. A genomic surveillance of SARS-CoV-2 and its variants was thus important. To this end, Austria employed an innovative approach termed SARSeq. We aimed to describe SARSeq and some noteworthy findings that it enabled on SARS-CoV-2 between January 2021 and March 2023.

**What have we learnt from this study?**

The SARSeq pipeline constituted a reliable and simple-to-implement surveillance tool. Genomic monitoring achieved through SARseq provided relevant insights on emergence of relevant SARS-CoV-2 variants in Austria. The surveillance also permitted faithful nowcasting and prediction of increases of COVID-19 cases (i.e. epidemic peaks). This information supported initiatives by public health authorities and bodies to mitigate the virus spread.

**What are the implications of your findings for public health?**

SARSeq is a resource-efficient genomic surveillance tool, not requiring complex automatisation. It was sufficient to detect multiple SARS-CoV-2 variants in Austria during the pandemic and to timely inform decision-makers on emerging variants. The method enables SARS-CoV-2 genomic surveillance beyond the pandemic and can be adapted to other pathogens. The pipeline can also be used in countries with limited technical resources and infrastructure.

## Introduction

During the COVID-19 pandemic, societies and global healthcare systems needed information on the number and extent of COVID-19 cases to identify disease transmission hotspots and implement targeted interventions. At the same time, severe acute respiratory syndrome coronavirus 2 (SARS-CoV-2) – the virus responsible for COVID-19 –, underwent rapid evolution, resulting in virus variants, some of which having increased transmissibility and/or enhanced capacity to escape host immunity. The appearance of variants underlined the value of complementing reliable COVID-19 case count estimates, with knowledge of genetic mutations appearing in circulating SARS-CoV-2 strains, so that viral infection trends in populations could be better understood and anticipated. The World Health Organization (WHO) and the European Centre for Disease Prevention and Control (ECDC) called for genomic monitoring and the European Commission recommended sequencing samples of at least 5% of SARS-CoV-2 cases [1].

Various strategies were developed worldwide to achieve useful and informative genomic surveillance for SARS-CoV-2 [2-10]. However, many countries fell short of meeting the challenging requirements of this objective. Moreover, since mid-2023, global sequencing capacities have dropped dramatically due to the high infrastructural, financial, and technical needs to maintain these [11,12]. As genomic surveillance also constitutes a pillar of pandemic preparedness, it must be sustainable, in other words, cost-effective, technically straightforward to implement and apply, as well as able to deliver rapid and high-quality results.

Here we present Austria's SARS-CoV-2 genomic surveillance pipeline, called SARSeq, which was used in the country during the COVID-19 pandemic. This pipeline relies on an approach developed in our laboratory, which uses focussed but highly multiplexed sequencing [13]. We aim to describe some aspects of the SARSeq setup and performance, that make it an attractive tool for baseline surveillance and to prepare for future pandemics.

## Methods

### Set up of a genomic surveillance in Austria

In winter 2020/21, the Austrian Agency of Health and Food Safety (AGES) expanded the comprehensive COVID-19 case-based surveillance with genomic monitoring of SARS-CoV-2. Collaborating laboratories provided SARS-CoV-2 RNA from specimens of cases in the nine Austrian provinces, as exemplified in the Supplementary Material Figure S11. AGES centrally collected and selected the RNA samples to obtain a representative number of these per province and arrayed them in multi-well plates. The required weekly sample size for variant detection at 1%, 2.5% or 4% was computed according to Wayne et al [14] and the ECDC technical report [15]. The Institute of Molecular Biotechnology of the Austrian Academy of Science (IMBA) obtained arrayed RNA from representative cases. RNA was processed to obtain genetic sequences using the SARSeq sequencing pipeline, followed by semi-automated analysis of the genomic data and weekly reporting of results.

### Principle of SARSeq

As opposed to adopting a whole genome sequencing (WGS) strategy, SARSeq focuses on amplifying and sequencing a genetic region of interest in the genome of a pathogen (e.g. the S-gene region of SARS-CoV-2). As in other protocols, amplification of this region of interest involves generation of multiple partially overlapping amplicons (i.e. tiles) that cover the whole region. The tiles are subsequently sequenced and sequence data obtained from each tile are then bioinformatically assembled revealing the entire region’s sequence. Through the sequence, some pathogen characteristics can be determined (e.g. a particular variant of SARS-CoV-2).

In conventional protocols, the PCR amplification process typically neither introduces sample identifiers that allow, when many cases are simultaneously investigated, to assign the pathogen’s sequence back to the infected individual, nor adapters for further steps such as sequencing. Thus, conventional protocols generally require several steps of ‘library preparation’ before the PCR, including end-repair, overhang generation and adapter ligation. These steps, and required clean-up procedures in between, can be costly, labour-intensive and require considerable automation. In contrast, the SARSeq strategy enables to add sample identifier indices and sequence adaptors directly during PCR amplification. SARSeq was adapted from a method that we originally developed for detecting SARS-CoV-2 and other viruses [13], as described in the Supplementary Material Supplement 1 section.

In the SARSeq method, many cases’ samples are simultaneously analysed. In brief, subsets of samples are assembled, in which tiles covering the genomic region of interest are amplified in parallel for each individual sample. Primers used to amplify the tiles all carry indices pointing to the individual sample. To prevent misassignments, the information encoded in the 5’ and 3’ primers is redundant, so-called ‘dual’ indices. Primers also contain common adapter sequences (i5, i7) for the next step. To enable encoding of multiple subsets, individual samples in a subset are then merged, so that each sample subset ends up in a sub-pool. A second PCR with complementary primers to the common adapter sequences (i5, i7) is then performed to further amplify all amplicons of each sub-pool in parallel. In this step, another set of indices, redundantly pointing towards the particular sub-pool are incorporated, as well as further sequencing adapter sequences (P5, P7). Together, this results in a combinatorial encoding of sample coordinates whereby each coordinate (i.e. sample and sample subset) is encoded redundantly.

We refer to this strategy as two-dimensional, unique dual indexing [13]. This design allows direct next generation sequencing (NGS) by Illumina platforms without the ligation-based library preparation of conventional protocols. The workflow reduces the entire process of sample preparation to six pipetting steps and circumvents intermediate purification steps, as presented in the Supplementary Material and in Supplementary Dataset 1. The multiplex nature of SARSeq, also enables it to obtain sequencing information from more than one genetic region of interest if more detailed characteristics of the pathogen are needed. Moreover, it also permits to obtain sequence data from several pathogens at the same time (e.g. SARS-CoV-2 and influenza A). More information on the SARSeq method can be found in the Supplementary Material and Supplementary Figure S2, as well as the Supplementary Datasets 1 and 2.

### SARS-CoV-2 genomic surveillance with SARSeq and expansion of the pipeline

For genomic surveillance of SARS-CoV-2 in Austria, the genetic region of interest was that encoding the N-terminal (NTD) and receptor-binding domains (RBD) of the spike protein. The region is shown in the Figure S1A of the Supplementary Material. The two domains are crucial for SARS-CoV-2 infectivity and human immune interactions [16,17]. The region comprises two-thirds of the S gene and ca 10% of the SARS-CoV-2 genome, yet from it all important information for variant assignment can be retrieved, as well as data to monitor new mutations of possible concern. This is illustrated in Figure S2A, B in the Supplementary Material. 

Amplicons of the tiles had a length of ca 280 bp. They had minimal overlap, except those covering the region for the RBD (encoding amino acid 470 to 502) to improve recovery of the RDB sequence, which is highly variable. To enable generation of desired amplicons, two independent sets of amplicons for this region were generated in parallel during the first PCR step. Subsequently, these sets were pooled. An illustration of the approach is provided in Figure S2C in the Supplementary Material. 

As the pandemic progressed, the SARSeq pipeline was successively expanded. More genetic regions of interest comprised the 5’ genomic region to detect recombinants (tiles 1 and 2), the nucleotide sequence for nonstructural protein 5 (nsp5) to monitor Paxlovid resistance (tile 3), as well as the N gene (tile 18). Amplification and sequencing of influenza A was also included to monitor SARS-CoV-2/influenza co-infections. The tiles in question can be seen in Figure S2B of the Supplementary Material.

Addition of an amplicon for the human gene encoding the β2 microglobulin protein (B2M) ensured monitoring of sample quality. The *B2M* gene was selected based on most reproducible detectability, as assessed by total RNA sequencing (RNAseq), and allowed the design of intron flanking primers to ensure RNA detection, unlike the commonly used, unspliced RNase P RNA control whose amplification is confounded by amplification of genomic DNA (Figure S2A, B in the Supplementary Material) [18]. The Supplementary Material presents more details in the Supplement 1 section, as well as all methods in the Data analysis subsection of the Supplement 2 section.

Successfully sequenced samples were included in the analyses. More information can be found in the Figures available in Supplementary Material and in Supplementary Table 1, where weeks with more than 30 samples per analysed province are displayed. 

### Assigning strains to lineages and mutation analysis

Analysis of sequence data was done semi-automatically. Upon completion of NGS, automatic demultiplexing, alignment, pangolin annotation as well as similarity-based clustering of sequences was conducted. For bioinformatic analysis and variant annotation an expert system, along with the pangolin annotation package was implemented [19], as explained in the Supplementary Material Supplement 1 and 2 sections. This was followed by manual sample annotation and case follow-up, as illustrated in Figures S2E and S5 of the Supplementary Material. Manual inspection was implemented due to the focused sequence coverage of the SARS-CoV-2 genome. This also allowed a good overview of rare or novel mutations. The automatic output additionally displayed non-fixed mutations (20–50% of reads for a sample) enabling detection of e.g. double infections by detection of mutations indicative of different SARS-CoV-2 sub-lineages, and viral subpopulations within patients (Figure S3A and B in the Supplementary Material). The bioinformatic script for semi-automatic analysis is available at GitHub (see code availability statement).

### Estimations of proportions of variants circulating

Extrapolation of relative variant proportions was based on weekly average of incidence counts from official case reporting by AGES, as shown in Supplementary Table 2, with sewage surveillance data used from calendar week (CW) 13 2022 onward, due to changes in testing behaviour. The conversion quotient was determined as the mean ratio of reported infections and sewage signal (https://abwassermonitoring.at/dashboard/) during the initial Omicron (Pango lineage designation B.1.1.529) wave (CW 09 2022−CW 22 2022). Details can be found in the Supplement S2 section ‘Extrapolated incidence’ of the Supplementary Material and the Supplementary Table 2.

### Nowcast

First, predicted absolute case numbers per variant were calculated based on observed growth rates from the previous 3 weeks. Subsequently a correction factor to real observed current COVID-19 counts was used to adjust e.g. for changes in social behaviour and population immunity. Details can be found in the Supplement S2 section ‘Nowcast’ of the Supplementary Material and in Supplementary Table 3.

### Doubling time and time to dominance

Doubling time for each variant was calculated based on curve fitting on total extrapolated incidences calculated as above. This is illustrated in Figure S14 of the Supplementary Material. Time to dominance (TTD) was calculated based on transition time from 10% to 60% prevalence on fitted sigmoidal curves as described in the Supplementary Material Figures S15A, S16. The Supplement S2 of the Supplementary Material and Supplementary Table 4 (sheet TTD) provide details.

## Results

### Efficiency of the Austrian surveillance system

SARSeq avoids conventional library-preparation steps, resulting in resource-efficient and streamlined processing with only six pipetting steps and no cleanup of samples. The pipeline enabled nationwide SARS-CoV-2 surveillance in Austria (ca 9 million inhabitants), with a team of two full-time employees and two half-time employees and simple robotic equipment, as shown in Figures S1–S9 in the Supplementary Material.

**Figure 1:**
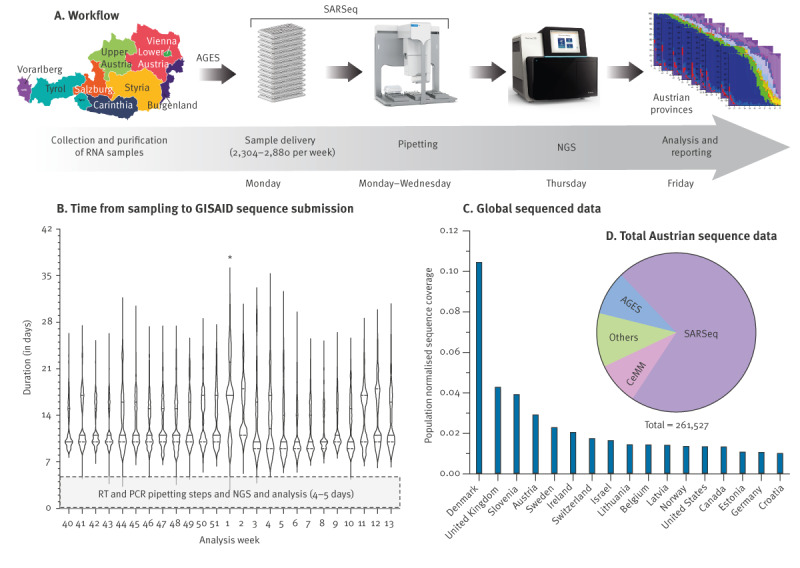
(A) Workflow and (B,C) efficiency of the Austrian SARS-CoV-2 genomic surveillance, as well as (D) relative contribution of SARSeq to the surveillance system, Austria, January 2021–March 2023

AGES: Austrian Agency of Health and Food Safety; CeMM: Research Center for Molecular Medicine of the Austrian Academy of Sciences; IMBA: Institute of Molecular Biotechnology of the Austrian Academy of Science; NGS: next generation sequencing; RT: reverse transcription.

Arrayed RNA samples were delivered to IMBA every Monday. The period of sample processing through the pipeline until results were obtained spanned 4 to 5 days, encompassing semi-automated pipetting from Monday to Wednesday, next generation sequencing (NGS) with automatic NGS data analysis (taking ca 5 hours), from Wednesday to Thursday, manual variant curation (details in ‘Data analysis and reporting’), and report preparation on Friday (Figure 1A).

A. Workflow of the Austrian genomic surveillance pipeline. RNAs from individuals are extracted by laboratories in the country’s provinces and AGES selects representative samples, which are delivered to IMBA where they are processed through SARSeq (pipetting for RT, PCRs) in preparation for NGS. After NGS, semi-automated analysis of sequence data enables reporting of the results.

B. Violin plots showing the duration in days from sample collection to sequence submission to GISAID per analysis week. The grey dotted box highlights the duration of RT, PCR, NGS and analysis. The asterisk (*) denotes a holiday-related analysis week, resulting in older processed samples due to pipeline inactivity.

C. Sequenced data obtained by Austria and other countries, in the period of SARSeq pipeline operation. Data are normalised to country population. Coverage was determined using information on amounts of generated sequences, available from GISAID (from the SARSeq period: 01.2021 to 03.2023, accessed April 2023), and population counts from Our World in Data (https://ourworldindata.org/grapher/population?time=latest; accessed April 2023).

D. Total sequenced data originating from Austria during the SARSeq pipeline operation.

The SARSeq-based Austrian genomic surveillance pipeline demonstrated a median turnover time of 11 days (minimum: 5 days; maximum: 34 days) from sample collection to sequence submission to GISAID (Figure 1B). The time from RNA sample delivery to GISAID sequence upload ranged from 4 to 5 days. The pipeline consistently performed without failures throughout its over 2-year operation and generated 72% of all sequenced data in Austria, ranking the country ninth globally in total sequence submissions and fourth (for countries with a population > 1 million) in terms of sequenced data per capita (Figure 1C,D; Figure S10 in the Supplementary Material and Supplementary Table 4).

### Output of the Austrian genomic surveillance

**Figure 2:**
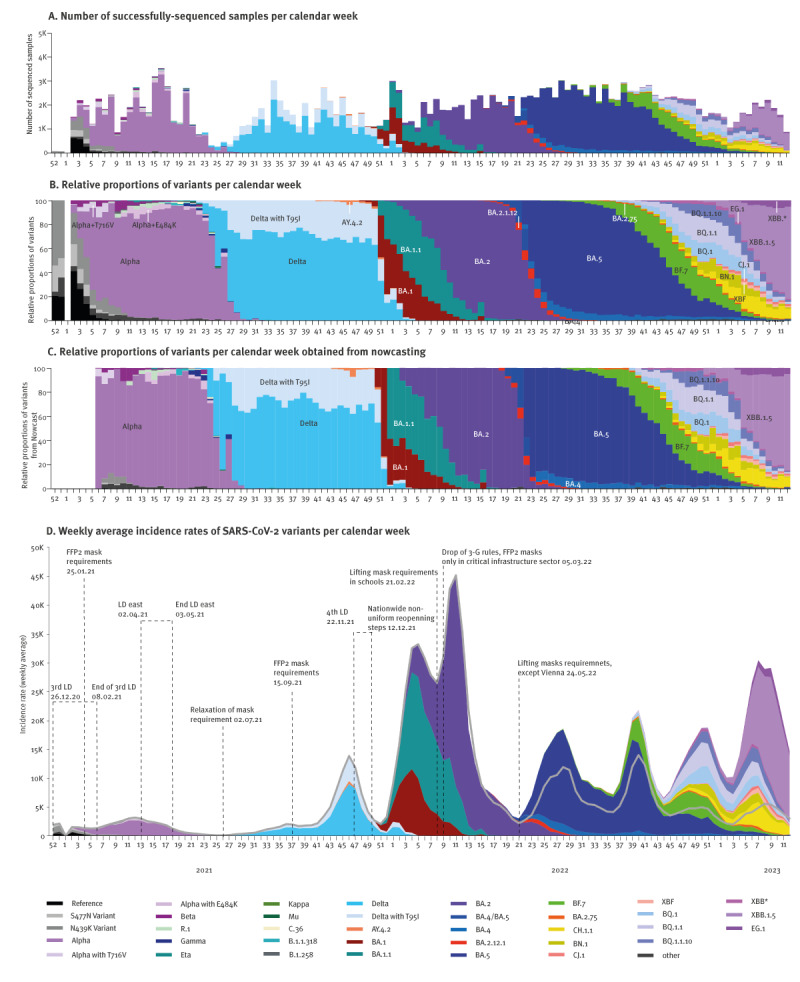
Integration of genomic data with epidemiological surveillance to monitor variant dynamics, Austria, January 2021−March 2023 (n = 222,784 samples)

The SARSeq pipeline started in January 2021 and successfully sequenced 222,784 samples by end March 2023 at an average rate of ca 2,000 samples per week (Figure 2A). This comprehensive dataset provided the opportunity to investigate the dynamics of multiple SARS-CoV-2 variants in Austria at province resolution, as shown in the Figure S13 panels A and B of the Supplementary Material.

Alpha: Pango lineage B.1.1.7; Beta: Pango lineage B.1.351; CW: calendar week; Delta: Pango lineage B.1.617.2; Eta: Pango lineage B.1.525; FFP2: filtering face-piece 2; Gamma: Pango lineage P.1; Kappa: Pango lineage B.1.617.1; LD: lockdown; Mu: Pango lineage B.1.621; Omicron parent lineage: B.1.1.529; SARS-CoV-2: severe acute respiratory syndrome coronavirus 2; XXB*: XBB and its sub-lineages; 3-G: ‘*Geimpft*, *Genesen*, *Getestet*‘, during certain periods of the COVID-19 pandemic in Austria admission to public places restricted to individuals vaccinated against SARS-CoV-2, recently testing negative for SARS-CoV-2 or convalescent after COVID-19.

A. Timeline of successfully sequenced samples per CW across Austria as a whole.

B. Timeline of relative proportions of variants per CW.

C. Computed nowcast data for each CW.

D. Integration of variant dynamics into epidemiological curves for Austria. Starting from CW13 2022, results were extrapolated using sewage data. The solid grey line represents officially reported case counts for comparison.

The methodology with semi-automated analysis also allowed us to identify various insertions of amino acids in spike position 212, which were detected 41 times in Austria; viruses with these insertions turned out to be prevalent in several European countries, yet overlooked by automated analysis tools [20]. We also detected co-infections with Influenza A in up to 3.8% of COVID-19 patients in December 2022 (Figure S3 in the Supplementary Material).

### Emergence of sub-lineages and mutations within specific variants

SARSeq not only allowed monitoring the emergence and spread of major lineages, but also tracking minor sub-lineages and significant single mutations over time, such as a Delta variant (Pango lineage designation B.1.617.2) with an additional spike T95I mutation, which emerged in Austria approximately 3 weeks after Delta and expanded with similar kinetics (Figure 2B and Supplementary Table 4, Mutations). While T95I was also observed in other variants (e.g. in AY.4.2 and BA.1), the ratio between both Delta variants remained largely unchanged, with approximately one third harbouring the T95I mutation.

Within the Alpha variant (Pango lineage designation B.1.1.7), diversification of the viral spike protein was observed, with the most prominent mutations being spike T716V and E484K (Supplementary Table 4, Mutations). Of greater relevance was the appearance of Omicron (Pango lineage designation B.1.1.529) sub-lineages BA.2 and BA.5 and recombinants during the second peak of the BA.5 wave, leading to a new ‘mixed’ wave. An obvious feature of this wave was a convergence of mutations such as spike R346, K444, V445, G446, L452, N460, F486, F490, through distinct phylogeny, due to natural selection for immune evasion against current predominant population immunity, as illustrated in Figure S12A in the Supplementary Material [21,22].

### Nowcast and its reliability

The pipeline’s key strength is its short turnaround time of just 4–5 days from RNA sample receipt or < 2 weeks from sample collection (Figure 1B). To predict the current variant mix at any given time we estimated the relative and absolute case number for each variant as nowcast. In hindsight, we evaluated the confidence of our predictions (Figure 2C). The maximal difference of the predicted variant share to observation was less than 5%, as shown in Figure S12C in the Supplementary Material and such discrepancies were only seen in situations of rapid variant take-over. Median accuracy of prediction was +/− 0.7%. For 81% of predictions, nowcasting predicted shares within 3% of measured value. Despite fluctuations in provincial representativeness, nowcasting was thus a precise tool to predict current variant mixes based on samples obtained within the 5–2 previous weeks and overcame lag-time included in the turnaround time of our pipeline.

### Weekly incidence estimates of the variants

Austria reported the highest per capita testing rate and consequently the highest per capita SARS-CoV-2 infections globally, as illustrated on the Supplementary Figure S13 panel D [23]. This allowed to inform models incorporating variant dynamics into epidemiological curves. When testing activities declined after BA.2, Austria had set up a detailed sewage monitoring system quantifying viral load in more than 90 wastewater treatment plants reaching an estimated 60% of the population [24]. We normalised viral load in sewage to case counts during the initial Omicron wave and modelled results based on sewage data from CW 13 2022 onwards. Displaying the variants in absolute numbers (Figure 2D) gave insight on their expected kinetics. On the panel D of this figure, officially reported case counts are shown as a solid grey line.

In several instances, variants re-emerged in response to relaxed measures or changes in social behaviours. The declining Delta wave experienced a short increase in CW 1 and CW 2 of 2022, likely linked to the end of the fourth lockdown and increased social interaction after the winter holidays. Similarly, the BA.1.1 variant showed a temporal increase in CWs 9 and 10 of 2022 likely due to the lifting of the mask requirement in schools and the dropping of 3G rules (admission to public places restricted to vaccinated, recently tested negative or convalescent individuals) in the same weeks. BA.5 increased again after the summer holidays in the first weeks of September 2022 (from CW 37 onwards).

Based on reported cases, Omicron BA.2 exhibited the highest total incidence numbers (1.58 × 10^6^ detected infections, 17% of the Austrian population) followed by BA.1 (including BA.1.1: 1.42 × 10^6^), and BA.5 (1.0 × 10^6^, Figure S12B in the Supplementary Material). However, sewage monitoring revealed that BA.5 accounted for most infections, with an estimated 1.72 × 10^6^ cases (19%).

### Consistent regional dynamics of major SARS-CoV-2 variants in Austria

**Figure 3:**
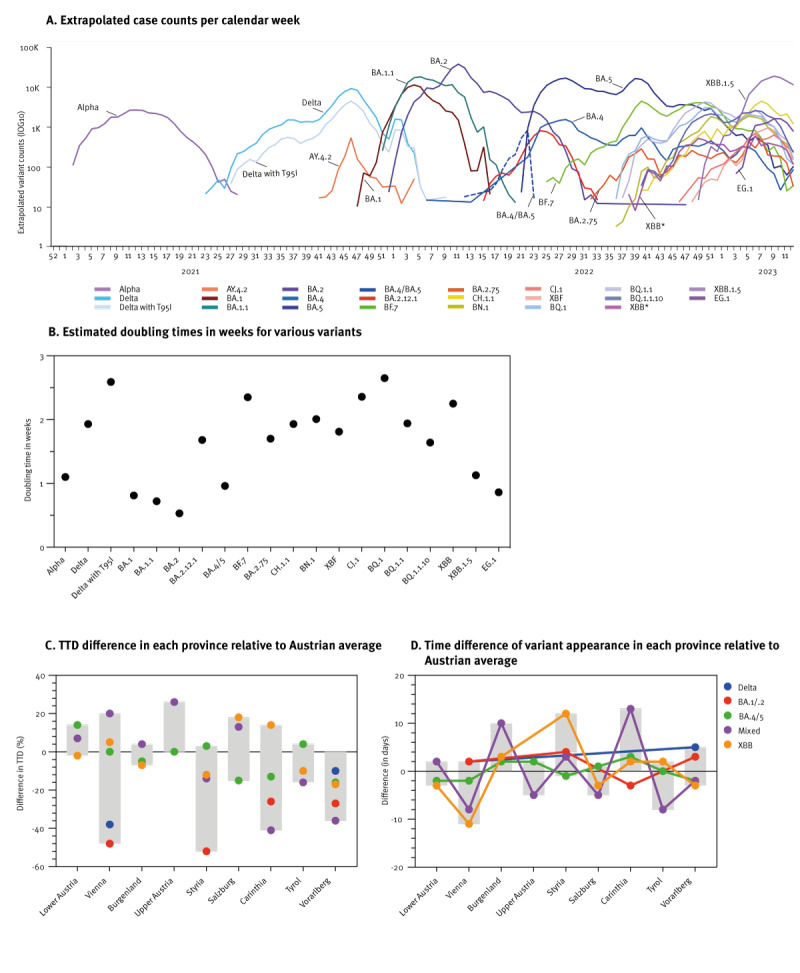
Dynamics of major SARS-CoV-2 variants in Austria and Austrian provinces, January 2021−March 2023

We aimed at quantifying the growth dynamics of SARS-CoV-2 variants in Austria. The distribution of extrapolated absolute case counts per CW illustrated initial exponential growth (as linear growth in the logarithmic scale, Figure 3A). We imputed doubling times of various variants by curve fitting, as shown in Figure S14 in the Supplementary Material. BA.2 cases doubled with a doubling time of 0.53 weeks, followed by BA.1.1 and BA.1 at 0.72 and 0.81 weeks respectively. Intermediate growth was observed for EG.1, BA.5, Alpha and XBB1.5 (0.86, 0.96, 1.10 and 1.13 weeks), while the doubling time of Delta, all BA.5 subvariants, as well BA.2.75 and its subvariants exceeded 1.5 weeks (Figure 3B).

Alpha: Pango lineage designation B.1.1.7; Delta: Pango lineage designation B.1.617.2; SARS-CoV-2: severe acute respiratory syndrome coronavirus 2; TTD: time to dominance.

A. Extrapolated absolute case counts from Figure 2D in the logarithmic scale to illustrate differences in the initial growth dynamics.

B. Calculated doubling times in weeks for various variants based on curve fitting (Figure S14 of Supplementary Material). BA.4 and BA.5 were analysed combined as BA.4/5, due to the limited number of samples for BA.4.

C. Per cent difference in TTD for each province compared with Austria. TTD is defined as the time from 10% to 60% prevalence using fitted curves (Figure S16 and Supplementary Table 4, TTD). The results are presented in days, with negative values indicating less time than Austria, and positive values indicating more time than Austria.

D. Variant appearance as defined by 10% prevalence in each province relative to the Austrian average based on the fitted curves (Figure S16 and Supplementary Table 4, Variant appearance). Negative values indicating an earlier appearance time and positive values indicating a later appearance time.

For analysis in C. and D. we defined variants with convergent mutations; BA.2.75, BN.1, CH.1, CJ.1, XBF, BQ.1, BQ.1.1, and BQ.1.1.10 as ‘mixed’ variants. Due to the limited number of samples for BA.1/BA.1.1/BA.2 as well as BA.4/BA.5 across all Austrian provinces, variants were analysed combined as BA.1/2 and BA.4/5 respectively.

Austrian provinces differ in geography, population density, and had distinct social distancing measures in place. We thus compared variant spread across Austrian provinces. Estimates of absolute growth were hampered by differences in testing (Supplementary Table 4, Test capacities AT provinces). We therefore compared relative growth rates of variants in the different provinces of Austria. All dominant variants showed remarkably parallel epidemiological curves across Austrian provinces except for Alpha that spread later in Tyrol than the rest of the country. The plots are presented in Figure S15A of the Supplementary Material.

To determine relative variant growth kinetics, the TTD of the variants was defined as the time from 10% to 60% prevalence using fitted curves (Figure S16 in the Supplementary Material and Methods). BA.1 exhibited the shortest TTD, followed by BA.5 and Delta. While BA.2 showed the shortest absolute doubling time (Figure 3B), its TTD was relatively long due to high prevalence of BA.1 when BA.2 appeared. Comparing Austrian provinces, we found that TTD depended primarily on viral variants, with little variation between provinces. We calculated the percentage difference in each province compared with the Austrian average. The province of Vorarlberg showed a trend of shorter TTD for all variants (10% and 40%, Figure 3C) while no trend emerged for other provinces. Vienna and Styria showed an almost 50% faster growth for BA.1/BA.2, but this translated only into 6–7 days. Of note, ‘mixed’ variants were not considered due to similar growth kinetics (Figure 3B), small relative shares, and sparse regional data. Overall, we observed a remarkable synchrony of variant expansion kinetics across all provinces, despite varying population densities ranging from 59 (Carinthia) to 4,654 (Vienna) inhabitants per square kilometre [25].

We next analysed variant appearance time, defined at 10% prevalence in each province relative to the Austrian average using fitted curves (Figure 3D and Figure S16 in the Supplementary Material). Overall, the appearance of variants was extremely homogeneous with time differences of typically < 1 week and followed no specific pattern. The co-existing ‘mixed’ variants exhibited the most substantial difference in reaching the first 10% prevalence and relative distribution differed between provinces. In contrast a minor difference was observed for BA.1/BA.2, BA.4/BA.5 and Delta variants. As prior noted, the only exception to this rule was the appearance of Alpha in Tyrol (Figure S15A in the Supplementary Material), that was delayed by approximately 1 month due to appearance of Beta (Pango lineage designation B.1.351) and accompanying testing programmes, travel restriction, and prioritised vaccine roll-out in this province [26,27]. Together, our analysis revealed surprisingly minor differences in the regional appearance and kinetics of the five dominant variants (Figure 3D), despite differences in geography and population density.

### Regional differences of minor variants in Austria

**Figure 4:**
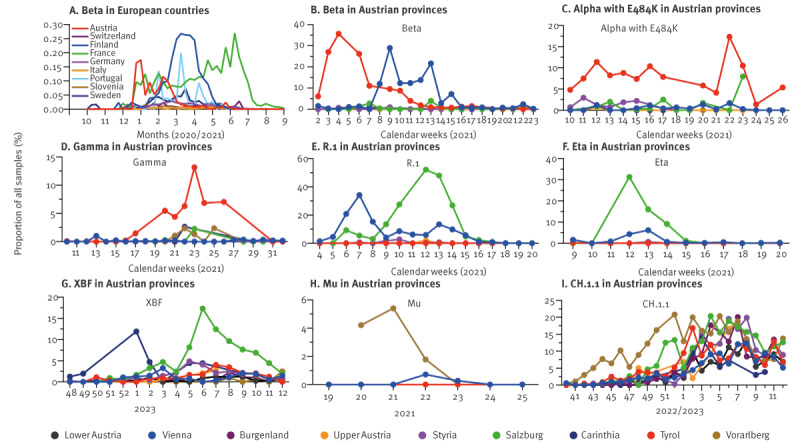
Prevalence during certain periods of minor SARS-CoV-2 variants in (A) European countries and (B−I) provinces in Austria, January 2021−March 2023

In contrast to major variants, we observed notable variations in the prevalence of some minor variants (Figure 4A-I). Of particular significance was the detection of a cluster of Beta in Tyrol, which occurred concurrently with the presence of the Alpha variant in Vienna and other provinces (Figure 4B and Figure S15A, Alpha, in the Supplementary Material), but not in Tyrol. Interestingly, the Beta wave in Tyrol preceded significant waves in other European countries (Figure 4A). Notably however, Beta rapidly declined in Tyrol while it persisted in Vienna and other European countries such as France, presumably due to effective containment measures in Tyrol specifically against this variant [26,27]. Following the Beta cluster, Alpha with a spike E484K mutation and Gamma (Pango lineage designation P.1) variants became predominant in Tyrol (Figure 4C,D).

Alpha: Pango lineage B.1.1.7; Beta: Pango lineage B.1.351; Eta: Pango lineage B.1.525; Gamma: Pango lineage P.1; Mu: Pango lineage B.1.621; SARS-CoV-2: severe acute respiratory syndrome coronavirus 2.

A. Prevalence of Beta variant across Europe.

B−I. Prevalence of minor variants across Austrian provinces.

The R.1 variant was detected in Vienna in February 2021 and briefly increased in prevalence while R.1 generated a larger cluster in Salzburg that was detected during ca 5 weeks in March 2021 (Figure 4E). Eta (Pango lineage designation: B.1.525; Figure 4F) and XBF (Figure 4G) also primarily expanded in Salzburg, while Mu (Pango lineage designation: B.1.621) was almost exclusively detected in Vorarlberg (Figure 4H), showing a decline in week 22 of 2022, preceding its designation as a variant of interest (VOI) by the WHO in week 35 [28]. CH.1.1 was also initially detected in Vorarlberg (Figure 4I). Remarkably most local clusters appeared in the western provinces of Austria that have more exchange with neighbouring countries and are geographically separated from the rest of Austria through mountain ranges. These observations highlight that numerous variants spread locally during the pandemic resulting in distinct infection histories of local populations and suggest that also today local variants exist worldwide − undetected due to sparse monitoring.

### Variant surveillance enables prediction of epidemic peaks 

**Figure 5:**
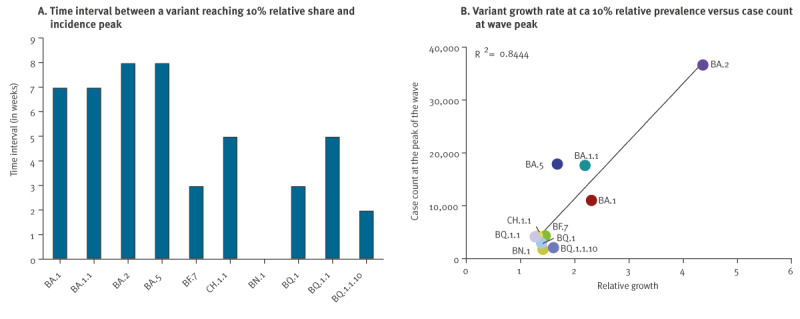
Monitoring SARS-CoV-2 variants enables the anticipation of epidemic peaks, Austria, January 2021−March 2023

During a pandemic, anticipation of incidence peaks is pivotal to timely break transmission chains and prevent a public healthcare system overload. To find out how early we could predict emerging variants with spread potential, we first retrospectively investigated if there was a correlation between a variant’s peak incidence and its prior growth rate observed at an incidence of 5% or 10%. Important variants reached this respective prevalence share at 7–9 weeks before incidence peak (Figure 5A and Figure S15B in the Supplementary Material) allowing for early prediction. Growth rates showed a very good correlation (R^2^ = 0.84 for 10% and 0.80 for 5% relative incidence) to the peak incidence detected later (Figure 5B and Figure S15C). Therefore, our surveillance system in Austria was sufficient to predict incidence peaks with potential to impact the healthcare system several weeks in advance.

SARS-CoV-2: severe acute respiratory syndrome coronavirus 2.

A. Time interval between variants reaching 10% relative share and incidence peak (Supplementary Table 4, variant growth and peak height).

B. Correlation between the relative variant growth at the time of reaching a prevalence of around 10% and the case count at the peak of the wave.

## Discussion

Our SARSeq-based sequencing and analysis approach demonstrated exceptional effectiveness and robustness to monitor SARS-CoV-2 throughout the COVID-19 pandemic. It ensured a comprehensive SARS-CoV-2 genomic surveillance in Austria, which ranked fourth globally among countries with over 1 million inhabitants in terms of sequencing intensity. With a total price of 20 EUR per sample in our pipeline, the setup was moreover resource-efficient. The methodology is adaptable and suitable for any sustained pathogen surveillance, and the protocol does not require complex automation. It is thus ideally suited for countries with limited resources as well.

The SARSeq protocol simplifies sample processing, also offering a fast turnaround time. SARSeq allowed sample processing, sequencing and annotation to be done consistently and without failure within 4–5 days, revealing, however, sample collection and shipment as bottle necks. The median analysis time from sample collection to sequence submission was 11 days (Figure 1B), with less than 50% of that time attributed to sample processing and sequencing. Improving logistics in sample collection and increasing the frequency of analysis runs could further maximise the timeliness of genomic surveillance.

The sampling strategy representative for time (week) and place (province) for SARS-CoV-2 genomic analyses across Austria enabled precise nowcasting (within +/ − 0.7%) aiding effective guidance to health interventions and response strategies in the country. Generally, this highlights the power of collaboration between state authorities, molecular biology and infectious disease epidemiology experts in academic settings.

Intensive person testing and sewage monitoring in Austria enabled a fine-grained extrapolation to absolute numbers of SARS-CoV-2 incidences as well as to estimate dynamics of variant spread. This analysis revealed a good correlation between initial growth rates and peak infection rates but not with the total infection count. Extrapolation to absolute numbers also showed the impact of reduced social distancing measures on the dynamics of already declining variants like a temporary re-appearance of Delta and BA.1.1, as well as the emergence of a second wave of the BA.5 variant.

Through SARSeq results, it was possible to observe that minor variants tended to spread locally, while major variants showed parallel kinetics across provinces. In Tyrol, the Beta variant was selectively tested for by rapid-turnover, variant-specific PCR in large testing efforts. Indeed, this variant dramatically declined in numbers shortly after its appearance but persisted for an extended time in Vienna and internationally. This shows that non-pharmaceutical intervention such as selective testing and isolation efforts focused on a specific variant can help to suppress its expansion, thereby changing relative fitness of the variant in the specific setting.

We further detected a notable co-infection rate of SARS-CoV-2 and influenza A in up to 3.8% of samples in week 51 of 2022. Based on official estimates [29], the influenza season 2022/23 peaked in weeks 50–51 with ca 5% of the population infected. While this finding suggests that there might be no strong evidence of viral interference between SARS-CoV-2 and influenza A, further investigations are required to thoroughly explore this aspect. This also demonstrates the possibility of expanding the panel of analysed respiratory infections to monitor rare or emerging pathogens both in a baseline surveillance setting as well as an immediate response to ‘disease X’ [30].

The strength of SARSeq is that it represents a middle ground between variant-specific qPCR and complete genomic analysis, providing a resource-efficient and effective alternative to WGS. On the other hand, the system has some limitations that should be mentioned. Concerning SARS-CoV-2 surveillance, the focus of SARSeq on the S gene somewhat limits sub-lineage resolution and recombinant detection. For example, we could not distinguish between BA.4 and BA.5, which vary in five non-silent mutations outside the S gene, in CW 17–22 of 2022 until we introduced tile 1 (Figure S2A). Similarly, we introduced tile 2 to identify the BA.1–BA.2 recombinant XE, as illustrated in Figure S2E in the Supplementary Material. The flexibility of the setup enables such updates seamlessly. Focused sequencing further hampered use of some automated phylogeny tools and was thus complemented with WGS, as shown in Figure S9B in the Supplementary Material.

## Conclusion

This retrospective analysis of SARSeq, a highly streamlined NGS pipeline for genomic surveillance of pathogens, illustrates its low constraints on automation, personnel, and costs as well as the flexibility to adjust to novel targets. With the detailed experimental protocols provided herein, SARSeq can serve as a blueprint to strengthen surveillance programmes globally.

## Supplementary Data

8813000 Supplementary Material

## Supplementary Data

379000 Supplementary Table1

## Supplementary Data

204000 Supplementary Table2

## Supplementary Data

462000 Supplementary Table3

## Supplementary Data

142000 Supplementary Table4

## Supplementary Data

3758000 Supplementary Table5

## Supplementary Data

48000 Supplementary Table6

## Supplementary Data

122000 Supplementary Dataset 1

## Supplementary Data

28000 Supplementary Dataset 2
