# Supplementary material for: Genomic surveillance of SARS-CoV-2 evolution by a centralised pipeline and weekly focused sequencing, Austria, January 2021 to March 2023
Source: Euro Surveill. 2024 Jun 6;29(23):2300542. doi: 10.2807/1560-7917.ES.2024.29.23.2300542 (PMC11158012; doi:10.2807/1560-7917.ES.2024.29.23.2300542)
Supplement: Supplementary Material [file 2300542_Supplementary_Material.pdf]

Supplementary Material

This supplementary material is hosted by Eurosurveillance as supporting information alongside the article [Genomic surveillance of SARS-CoV-2 evolution by a centralised pipeline and weekly focused sequencing, Austria, January 2021 to March 2023], on behalf of the authors, who remain responsible for the accuracy and appropriateness of the content. The same standards for ethics, copyright, attributions and permissions as for the article apply. Supplements are not edited by Eurosurveillance and the journal is not responsible for the maintenance of any links or email addresses provided therein.

Supplement S1. Supplementary method development and benchmarking

Design of a robust, scalable and cost-effective mutation surveillance pipeline

Mutational surveillance to detect changes in the pathogens genome during a pandemic demands solutions that are both robust and simple to implement, but also scalable and cost-effective. Sequencing the genome of the pathogen of interest using NGS offers an optimal approach for standardized high-throughput analysis. However, several challenges must be met: i) the sensitivity of the analysis approach must be sufficient to deliver results across the range of viral titers observed in positive patients; ii) reliable scalability to thousands of patient samples as well as multiple amplicons per sample in parallel; iii) short turnaround time for sequencing results to empower case-based follow-up measures; iv) rapid setup of operations to enable quick responses; v) costs must be kept as low as possible as they might otherwise be prohibitory for large sample sizes. We sought to develop a simple and fast workflow for SARS-CoV-2 S gene sequencing that overcomes these challenges and enables the analysis of thousands of samples in parallel by tackling the rate-limiting steps of current WGS protocols: i) eliminating the need for labor- and time- intense NGS library preparation downstream of PCR amplification and ii) reducing sequencing time iii) increasing the scalability to thousands of samples in parallel and iv) reducing costs. We started by designing a set of PCR amplicons that tile the desired portion of the S gene (**Supplementary Figure S2A, B**). The widely used ARTIC design for SARS-CoV-2 tiles the whole genome with ~400 bp amplicons to which sequencing compatible adapters are added in a library preparation step, with a resulting library that must be sequenced using a 500-cycle kit, typically in paired-end 250 modality (PE250) in case of Illumina platform[1,2]. We aimed to design a sequencing approach around a PE150 sequencing modality with amplicons of up to 281 bp, excluding adapters, to reduce costs, improve sequence quality, and increase sequencing speed for faster turnaround time and higher sequencing throughput per machine. This approach reduces sequencing time by about 12 hours and costs by EUR 1400/run in our setting. The PE150 modality further avoids competition with the standard WGS pipeline for potentially limiting reagents. Amplicons were partially overlapping in order to cover the whole region of interest, yet we minimized this overlap for most amplicons. A notable exception is the most variable region of the RBD [3] from amino acid 470 to 502, where a 105 bp overlap was incorporated in order to cover this critical region, which harbors several known mutations of concern, with two independent amplicons (**Supplementary Figure S1**). This design resulted in 14 amplicons that tile the N-terminal part of the SARS-CoV-2 **(Supplementary Figure S1B)** [4–6], thus allowing us to detect mutations affecting the protein surface with duplicate coverage of the most flexible region of the RBD.

To implement a simple, cheap and high-throughput workflow for amplification and sequencing we adapted SARSeq, a method we recently developed for detection of SARS-CoV-2 and other viruses [7]. SARSeq is a robust pipeline to reverse transcribe and amplify multiple nucleic acid segments in parallel, and across thousands of individual samples, while maintaining high sensitivity and specificity. SARSeq uses a two-dimensional, unique dual indexing strategy, in which every sample is barcoded directly in two subsequent PCR reactions with 4 indices in total. By also incorporating all required adapter sequences (i5, i7, p5, p7) in these steps, the PCR products generated by SARSeq can be directly sequenced by Illumina platforms, without the need for further library preparation steps [7]. This workflow reduces the entire process to 6 pipetting steps where reagents are added or pooled and does not require any intermediate purification step (**Supplementary Dataset 1 and Supplement S2**). In brief, these steps are i) addition of sample to the RT mix, ii) addition of PCR1 mix to the RT reaction where sample-specific indices are added, iii) pooling, iv) addition of ExoProStar to remove unused primers, v) addition of PCR2 mix where a second set of indices for combinatorial barcoding are added, as well as adaptors for Illumina sequencing, and vi) final pooling.

To achieve sensitive, robust and uniform amplification across the S gene, multiple experimental parameters had to be adapted relative to the original setup [7]. First, because overlapping amplicons cannot be produced within the same reaction, we split the "Blue" and "Red" amplicons or tiles in two independent RT- and PCR reactions using the respective primers for each set (**Supplementary Figure S2A and B**), as is also done for whole genome sequencing using the ARTIC primer design [2]. We called this strategy SARSeq S-tiling. For each sample, both the "Blue" and "Red" PCR products receive the same index pairs. Second, we found that using a specific RT primer with a melting temperature of ~58ºC for each amplicon, as opposed to conventional random priming using hexamers, resulted in markedly increased read counts for all amplicons (**Supplementary Figure S4A and B,** Pipeline optimization was initially done with 13 tiles covering the S-gene. An additional tile was included later during the operational period). In particular for Tile 10, that

reproducibly gave the lowest read counts, the addition of a second tile-specific RT primer made a significant difference (**Supplementary Figure S4C**). We assume that the longer gene-specific primers can still bind the RNA template at the RT temperature of 55ºC, as opposed to hexamers, and thus support reverse transcription in regions with higher degree of secondary structure. Third, RT- and PCR protocols were adapted to suit the longer amplicons and suppress generation of short, unspecific amplicons (**Supplementary Dataset 1 and Supplement S2**). Fourth, RT- and PCR primer concentrations were optimized to balance coverage across tiles. Last, the general pooling scheme and setup of PCR2 was adapted to the use of many parallel amplicons (**Supplement S2**).Together these adaptations yielded a robust PCR pipeline that successfully amplified all tiles in a multiplexed PCR reaction.

Measures to ensure quality and specificity

When processing thousands of positive samples, cross-contamination is a major concern. We implemented the following measures to prevent sample cross-contamination: i) strict local separation of pre- and post-PCR steps, ii) addition of dUTP to both PCR reactions as well as UDG prior to starting PCR1 in order to degrade previously amplified, contaminating DNA molecules, iii) use of distinct PCR1 indices in neighboring pools, iv) use of alternating PCR2 indices each week, and v) strict monitoring of undesired amplification products.

To filter against spurious reads generated by index hopping [8,9], we implemented a cutoff for each tile, whereby we demanded at least 1% of the median read number of the respective tile in the run (or a minimum of 10 reads). This cutoff correctly eliminated spurious signal in 98.6% of presumed empty wells. The defined per-tile cutoff was also used to derive an overall sample filter that reflected the overall quality of a sample: samples with fewer than 6/13 tiles above cutoff, were considered of insufficient quality and were not subsequently analyzed for variants.

Pipeline sensitivity and scalability

Positive samples for SARS-CoV-2 typically span a large range of viral titers, with differences of up to six orders of magnitude [10]. If multiple samples are to be sequenced in a highly parallelized setting, each sample should produce read numbers where the range of 10^6 is condensed to 1-2 orders of magnitude at most to avoid that high titer samples are over-represented and lower titer ones missed. To dampen this difference, we allowed PCR1 (on individual samples) to reach end-point. This blunted original differences in titer sufficiently to maximize the use of available sequencing depth. The ability to obtain sequencing data even from low titer or bad quality samples is an important feature of such a surveillance pipeline. To assess sensitivity directly, we correlated SARSeq S-tiling performance to Ct values measured with a commercial kit on the N1 amplicon [11] for a set of 192 clinical samples (**Supplementary Figure S6A**). We found that all amplicons could be detected for 93% and 89% of samples at below Ct=33 and 35 respectively, which corresponds to <10 molecules per reaction [7]. The redundantly covered variable region of the RBD was detected in 99% of samples at these cutoffs (**Supplementary Figure S6B**). The RT-PCR protocol employing SARSeq S-tiling is thus efficient to amplify all tiles at similar sensitivity down to a few molecules per sample.

The sensitivity of any sequencing pipeline is inversely proportional to the number of samples that are pooled in one sequencing run. We therefore wanted to estimate how many more samples we could pool in one sequencing lane in the current setup (one NovaSeq lane, about one billion reads) and still maintain a similar level of sensitivity. To this end we down-sampled the reads from a typical run by a factor of ten and plotted the number of detected amplicons while maintaining the established cutoffs. Of the samples with Ct values <33 and <35 we could still detect at least 12/13 amplicons for 91% and 86% respectively (**Supplementary Figure S6C**). Tile 10, which produces the lowest number of reads, was still detected in 77% and 70%, and importantly, the variable region of the RBD was still covered for 99% of samples. Thus, the robust performance of the pipeline should allow for multiplexed sequencing of up to 23,000 patient samples with Ct<33 together in a single NovaSeq lane with a target output of 1 billion reads. This would bring sequencing costs to a few cents per sample and highlights the cost-effective and scalable nature of SARSeq.

Setup of a surveillance pipeline for Austria

To set up nationwide mutational surveillance of SARS-CoV-2 in Austria we established a pipeline that would process up to thirty 96-well plates weekly with a single NovaSeq PE150 run. To guarantee delivery of robust sequencing results we developed a standard operating procedure (**Supplementary Dataset 2**) including robotic assisted pipetting, an optimized NGS read mode, and a hand-over protocol to initiate NGS analysis automatically upon completion of sequencing. We processed up to 30 x 96-well plates of positive samples, namely up to 2,880 wells per week with a team of two full-time and two part-time members and delivered SARS-CoV-2 spike gene sequences from RNA preparations in the period of January 2021 – end March 2023. Each processing cycle was initiated on Monday and sequencing data were delivered on Friday of the same week. To aid rapid bioinformatic analysis, we implemented an expert system for semi-automated and assisted manual annotation. In order to detect and quantify known and novel sequence variants of the SARS-CoV-2 S gene, the bioinformatic analysis was performed in several steps: i) the paired-end reads were assigned to sample IDs via the two-dimensional dual indexing as previously described [7]; ii) the reads of each sample were assigned to the intended tile via the corresponding primer sequences at the ends of each amplicon (invalid amplicons were discarded); iii) the amplicon sequences without the primers were aligned to the respective segments of the SARS-CoV-2 S-gene reference using a strategy that allowed for mismatches during alignment, and such variants were subsequently detected and annotated at the DNA and protein level (**Supplement S2 and github link**). Results are output as spreadsheet tables for manual inspection and annotation with each row showing one sample and each column

showing one nucleotide or amino acid. Reference positions are denoted as "+" and mutant positions as the respective nucleotide or amino acid symbol. We also automatically generate a simplified version of the output table that only shows amino acids that differ from the reference and groups the samples by similarity of mutational signature. To facilitate variant annotation, samples are clustered based on mutational signatures (**Supplementary Figure S2E**). In addition, for each sample we provide the numbers and percentages of mapped reads per tile as well as other metrics to assess sample quality. The complete bioinformatic analysis takes ~5 hours to process 1 billion reads and up to 2,880 samples, producing an ideal output for manual sample annotation and case follow up.

For the Austrian surveillance pipeline, SARS-CoV-2 positive samples were delivered to us upon RNA purification as separate 96-well plates by labs throughout Austria. Despite deemed positive, the samples spanned the natural range of viral RNA titers and even included empty wells and (partially) degraded samples or with little remaining volume. We evaluated the performance of SARSeq S-tiling on ~25,000 samples, processed in eleven independent runs. We observed that all tiles were relatively uniformly covered in ~80% of samples, using the cutoffs described above (**Supplementary Figure S6D**). Failed tiles were not uniformly distributed across samples, but rather, samples tended to either produce reads for all tiles or for none (**Supplementary Figure S6E**), likely indicating low level, low quality or absence of any template RNA in ~20% of the samples. This is consistent with the amplicon-coverage for samples with low viral titers (**Supplementary Figure S6A, C**) and suggests that in a real-world setting, up to 20% of PCR-positive samples collected from various diagnostic labs are not compatible with sequencing.

We scaled these data to mimic the pooling of 23,000 samples per run (**Supplementary Figure S6F**). This would have reduced the performance of amplicons 4, 7, and 10 but still maintained a robust pipeline delivering sequence information for relevant regions of the S gene. SARSeq based S-gene tiling is thus sensitive to a Ct value of 35 and reaches good performance on real-life clinical samples across multiple sample providers.

Pipeline Reproducibility

The next aspect we explored is the accuracy of the sequence information derived by the pipeline at the cutoffs we had set. To test reproducibility of our sequencing calls, we took advantage of the redundancy built in by our primer design. Specifically, amino acids 470-502 are covered by amplicons 11 and 12, which are processed in two independent reactions, the "Red" and "Blue" batch respectively. As multiple samples carry amino acid substitutions in positions S477, E484, and N501, we compared the calls for these positions from tile 11 and tile 12 for >10,000 samples. We found a near perfect agreement for samples where data was available from both amplicons (**Supplementary Figure S7A**). Both the reference sequence as well as mutations are called with extremely high reproducibility. Only two cases where observed, where a high confidence mutation is not confirmed in the other amplicon and might represent a PCR- or sequencing error. The error rate is thus 2 out of ~30,000 or <0.01% per amino acid. Mutation calls with low confidence (from read numbers between 10 and the median-based read cutoff) and/or sub-stochiometric representation within the sample (i.e. changes reported in lowercase) show a slightly lower coverage reproducibility but no discrepancy between results. The error rate we calculated estimates that ~1/20 samples will have one error across the covered ~700 amino acids. We therefore placed an additional cutoff, and only report amino acid changes when they occur in three or more samples within each batch of up to 2,880 samples we process together. The probability that identical changes in any amino acid occur with this frequency is infinitesimal, thus we chose this stringent cutoff to report variant positions. SARSeq S-tiling therefore generates high-confidence sequence analysis of most of the SARS-CoV-2 S gene at low cost and high throughput.

We finally challenged the sensitivity and robustness of the pipeline to call mutations present at low frequency within a single sample. To do this, we selected two variants that differ in 10/13 tiles, namely Beta and Alpha harboring an additional S13T mutation (**Supplementary Figure S7B**). Purified RNA from patient samples was mixed at different ratios and a dilution series was generated from these mixes to a Ct range of 24-35 as confirmed by RT-qPCR (**Supplementary Figure S7C**). Samples were then subjected to SARSeq S-tiling alongside a batch of 2,300 other samples (**Supplementary Figure S7D, E**). For every condition, we extracted the percentage of each of the two variants in every individual tile. SARSeq S-tiling quantitatively retrieved the fraction of each variant in the respective mixes. Concordance across tiles was high even for the more diluted samples, with standard deviations between amplicons in equimolar mixes ranging from 7% at Ct=24 to 28% at Ct=35. SARSeq S-tiling was even able to robustly quantify a spike-in of 1% of a virus strain in the background of a different strain. Whereas this was beyond the goal of our study and our surveillance responsibilities, such low-frequency mutations were indeed frequently observed and can be used to gain further insight into lineage relationships between sequenced samples [11]. In summary, SARSeq S-tiling delivers qualitatively and quantitatively reproducible sequence across the ectodomain-coding region of the S-gene, that contains most mutations currently considered to be biologically impactful.

Supplement S2 Supplementary Materials and Methods

RNA purification

Left-over, purified RNA samples from SARS-CoV-2 positive samples was provided by the Austrian Agency for Health and Food Safety (AGES) and remained fully anonymized to us. These had been purified by various laboratories using different purification pipelines and were shipped regularly.

Reverse transcription

For each sample, RT and and PCR1 are performed in two independent reactions, one for "Blue" amplicons and second for "Red" amplicons. Per sample 5 µl of RNA sample was mixed with 20 µl of reverse transcription reaction master mix. RT reaction master mix is prepared by combining 13.8 µl of nuclease free water, 2.5 µl of 10X RT buffer, 0.5 µl of dNTPs (25 mM each), 0.1 µl of 1M DTT, 2 µl of RT primer mix ("Blue" set contains 13 primers and "Red" set contains 10 primers. Sequences of RT primers can be found **in Supplementary Dataset 1**), 0.5 µl of Ribonuclease inhibitor and 0.5 µl Reverse transcriptase. RT mix was arrayed out in 96 well plates, stored on ice, and reactions were started within 1h. The RNA sample and RT reaction master mix combination was mixed 10 times by the liquid handling robot and incubated in a humid incubator at 55ºC for 40 min. Subsequently the reverse transcriptase was inactivated at 95ºC for 3 min and the reaction cooled down to 4ºC. **Supplementary Dataset 1** provide detailed concentrations and calculations on volumes of the pipetting scheme. For regular analysis components were premixed and frozen in aliquots such that upon addition of 15ml ddH_2O and enzymes the mix was ready for 12 x 96-well plates (see **Supplementary Dataset 2).**

10× RT Buffer:

200 mM Tris-HCl pH 8.3

500 mM KCl

50 mM $MgCl_2$

200 mM $(NH_4)_2SO_4$

1% Triton X-100

First PCR (sample indexing)

The PCR top-up master mix was prepared by combining 11.43 µl of nuclease free water, 2.5 µl of 10X PCR top-up buffer, 0.07 µl of dUTP (100 mM stock), 0.5 µl of Antarctic thermolabile UDG, 0.5 µl Hotstart Taq Polymerase and 10 µl of PCR primer mix with sample specific unique dual indexes ("Blue" set contains 10 primer pairs/1 µM each OR "Red" set contains 10 primer primers/1 µM each, sequences of PCR primers can be found in **Supplementary Dataset 1**). 25 µl of PCR top-up master mix per reaction is added and mixed ten times with the liquid handling robot. The reaction should be kept on ice. The mixed reaction is removed from ice, kept at RT for 5 min to allow reaction of the UDG, and put into a thermocycler that is already at 95ºC (hot start). The program is: 95ºC for 3 min, 10 high stringency PCR cycles (95ºC for 30 sec and 63ºC for 5 min) followed by 35 high efficiency cycles (95ºC for 30 sec, 63ºC for 30 sec and 72ºC for 40 sec), then cool down to 12ºC. Successful amplification of S-gene specific amplicons (~330bp) can be analyzed by resolving 10 µl of PCR reaction on a 2% agarose gel.

A master mix containing all components listed below, including homemade HotStart Taq Polymerase and Uracil DNA glycosylase (Antarctic Thermolabile UDG from NEB) was prepared and distributed to a 96-well plate containing previously arrayed primer pairs (multiple primer plates can be prepared simultaneously and stored frozen at -20ºC). Using a liquid-handling robot, the primers and PCR master mix were mixed thoroughly and 25 µL of this complete 2× PCR mix were added to the 25 µL RT reactions ran as described above. Plates were sealed with aluminum sealing foil and incubated in a thermocycler following the conditions listed below.

All components were kept at room temperature during reaction set up and for 5 min before starting the PCR program, this provides the right conditions for UDG to act on Uracil-containing amplification products of previous PCR reactions, thereby removing spurious carry over contaminants. After UDG heat inactivation, the subsequent PCR reaction was again carried out in the presence of UTP to prevent carry over contamination in following runs. **Supplementary Data 1** provide detailed concentrations and calculations on volumes of the pipetting scheme. For regular analysis components were premixed and frozen in aliquots such that upon addition of 15 ml ddH_2O and enzymes the mix was ready for 12 x 96well plates (see Supplementary Data 3).

10× PCR Top Up Buffer:

750 mM Tris-HCl pH 8.3

200 mM $(NH_4)_2SO_4$

1% Triton X-100

Plate pooling

All well-barcoded PCR products from each row of a 96-well plate were pooled, typically 10 µL of each reaction were combined in an 8-tube PCR strip using a multi-channel pipette, and after mixing thoroughly, 50 µl of each the "Blue" and "Red" pooled mixes were transferred to a new 96-well plate (see schematic in **Supplementary Dataset 2**). This was repeated for every pair of PCR plates. 5 µL from each plate pool were re-arrayed in a new 96-well plate and treated with 2 µL of illustra ExoProStar 1-step for 30min at 37ºC followed by 15 min at 80ºC to remove any left-over primer.

Second PCR (plate indexing and addition of sequencing adaptors)

A master mix with all components listed below was distributed across a 96-well plate (40 µL/well). To each we added 7.5 µL of unique dual-indexed i5/i7 primer pairs (Custom synthesized index primers with Nextflex barcodes, arrayed in 96-well plates) and 2.5 µL of ExoProStar-treated PCR1 pool. The reactions were run for 8 cycles to add sequencing adaptors with plate barcodes. 2.5 µL of ExoProStar-treated PCR1 pool are transferred into a 96-well plate containing and array of unique dual-indexed i5/i7 primer pairs (7,5ul each, custom synthesized index primers with Nextflex barcodes). To avoid cross contamination from previous runs indices used for PCR 2 were alternated between weeks. A master mix with all components listed below was added to each well. The reactions were run for 8 cycles to add sequencing adaptors with plate barcodes.

10x Sequencing-ready PCR Buffer:

750 mM Tris-HCl pH 8.3

200 mM $(NH_4)_2SO_4$

20 mM $MgCl_2$

0.1% Tween 20

Master mix composition per reaction/well (volumes in µL):

10× Sequencing-ready PCR Buffer	5
25 mM each dNTPs	0.5
100 mM dUTP	0.07
Hotstart Taq Polymerase	0.5
Water	33.93

Thermocycler program:

3 min at 95ºC

8 cycles of: 30 sec at 95ºC, 30 sec at 65ºC, 40 sec at 72ºC

3 min at 72ºC

Cool down to 12ºC

Final Pooling and preparation for sequencing

All samples from a 96-well plate (20µl from each well) were pooled and 200µl of pooled sample was resolved on a 1,5% agarose gel and 400-500 bp amplicons were excised and gel purified using Qiagen gel extraction kit.

Sequencing

Quality control of libraries was performed in the fast, optimized manner described previously [6]. Libraries were sequenced on a NovaSeq6000 SP flowcell with a loading concentration of 220 pM and a custom read mode of 156/9/9/164 (read 1/index1/index2/read2). To increase the complexity of the sequencing sample, 10 % PhiX were spiked-in in every run. Due to the combinatorial two-dimension barcoding of the libraries, bleach washing steps on the NovaSeq can be omitted, because DNA remnants, potentially present in the sequencer's fluidics from previous runs, can be filtered out. Nevertheless, a bleach wash was performed every 14 days.

Data analysis

Paired-end (PE) reads were assigned to sample IDs using the two-dimensional dual indexing as described (SARSseq [6]). Subsequently, the PE reads of each sample were further assigned to amplicons/tiles based on the respective amplicon/tile-specific primer sequences that are contained within the reads using a custom awk script, enabling the specific alignment of each amplicon/tile. The PE-read-to-tile alignment was performed using minimap v2.17 and the SARS-CoF-2 reference sequence MN908947.3 from which we extracted the corresponding tile/amplicon reference

sequences. After alignment, we retained only reads that mapped in proper pairs using samtools v1.10. a textual pileup of the filtered alignment was obtained using samtools mpileup 1.10 using parameters "-aa --max-depth 0 --no-BAQ --min-MQ 1" and further summarized using readstomper.pl and R v3.6.3. We derived a sample-specific S gene consensus sequence from the pileup and translated this consensus sequence into the corresponding S protein sequence. The consensus encoding was based on two cutoffs- a minimal read cutoff (mRC) of 10 and a per tile confident coverage cutoff (cRC) defined as 1% of the median observed coverage of that tile in the respective experiment. Using these cutoffs we created an annotated consensus sequence highlighting variants to the reference according to the following rules: "": read coverage < mRC; "_": read coverage < cRC and no variant allele above 0.5; "lower case" or ".": read coverage < cRC and variant allele/deletion above 0.5 or confident read coverage and variant allele observed in 0.2-0.5 fraction of reads; "upper case" or "-": variant allele or deletion with confident read coverage.

Quantitative RT PCR (qPCR) assay

The RT was performed as described in the reverse transcription section, using 5 µl of sample in 25 µl RT reaction. For qPCR analysis, 25 µl of PCR1 top up reaction mix with 1.5 µl of CDC-N1 primer/probe set (IDT 10006713/sub part 10006600) was added to the completed 25 µl RT reaction mix. Reactions were run at 95°C for 3 minutes, followed by 45 cycles of 95°C for 15 seconds and 55°C for 45 seconds in a BioRad CFX Connect™ Real-Time System.

Generation of figures

Statistical analysis and plots were done using GraphPad Prism 8.4.3., plate layouts were illustrated in Microsoft Excel, and figures were assembled in Adobe Illustrator CS6.

Code availability statement

Custom code was used to analyze all NGS data. The script is available at GitHub under https://github.com/sarseq/sarseq2.

Extrapolated incidence

To estimate the incidence of each variant, we extrapolated each variant's relative proportion using weekly average of incidence counts (Monday to Sunday) based on official case reporting by AGES (see **Supplementary Table 2)**. Due to gradual changes in testing behavior, we based incidence estimates on sewage surveillance from CW13 2022 onward. The conversion quotient was based on a median ratio of reported infections and sewage signal normalized to human contribution (**https://abwassermonitoring.at/dashboard/**) during the initial Omicron wave (CW09 2022-CW22 2022). For details see supplement (**Supplementary Table 2**).

Nowcast

To estimate the relative proportion of a certain variant for the week of report, we used a two-step process. First, we calculated the relative growth for each variant using the extrapolated total numbers for each week based on the number of cases reported to the Austrian comprehensive laboratory-based SARS-CoV-2 surveillance systems. Next, we computed the geometric mean (GM) of the relative growth over the past three weeks, multiplied this GM with the extrapolated data from the previous calendar week to generate the nowcast for the particular calendar week of report. Secondly, to increasing the accuracy of our nowcast estimate and account for recent changes in social behaviour, we multiplied the estimate by a correction factor. This factor was obtained by dividing the predicted actual incidence (PAI) cases for the analysis week by the sum predicted case counts for the same week based on variant growth. To calculate the PAI in a given (incomplete) analysis week, while blunting reporting variation between different days of the week, we averaged the ratio of officially reported case numbers on Monday through Wednesday relative to the respective value in the week before and multiplied it with the average case number of the last seven days. For details see supplement (**Supplementary Table 3).**

Doubling time and time to dominance

Doubling time for each variant was calculated based on curve fitting on total extrapolated incidences calculated as above (**Supplementary Figure S10 and Supplementary Table 4)**. BA.4 and BA.4/.5 case numbers were combined with BA.5 for this analysis. To calculate time to dominance (TTD) for each province, we plotted the fraction of dominant variants per week (**Supplementary Figure S11**). To determine TTD of the variants, we fitted sigmoidal curves in the growth phase (**Supplementary Figure S12**) and assessed transition time from 10% to 60% prevalence (**Supplementary Table 4)**. For this analysis we defined variants with convergent mutations; BA.2.75, BN.1, CH.1, CJ.1, XBF, BQ.1, BQ.1.1, and BQ.1.1.10 as "Mixed" variants. Due to the limited number of samples for BA.1/BA.1.1/BA.2 as well as BA.4/BA.5 across all Austrian provinces, variants were analyzed combined as BA.1/2 and BA.4/5 respectively.

Supplementary Figures

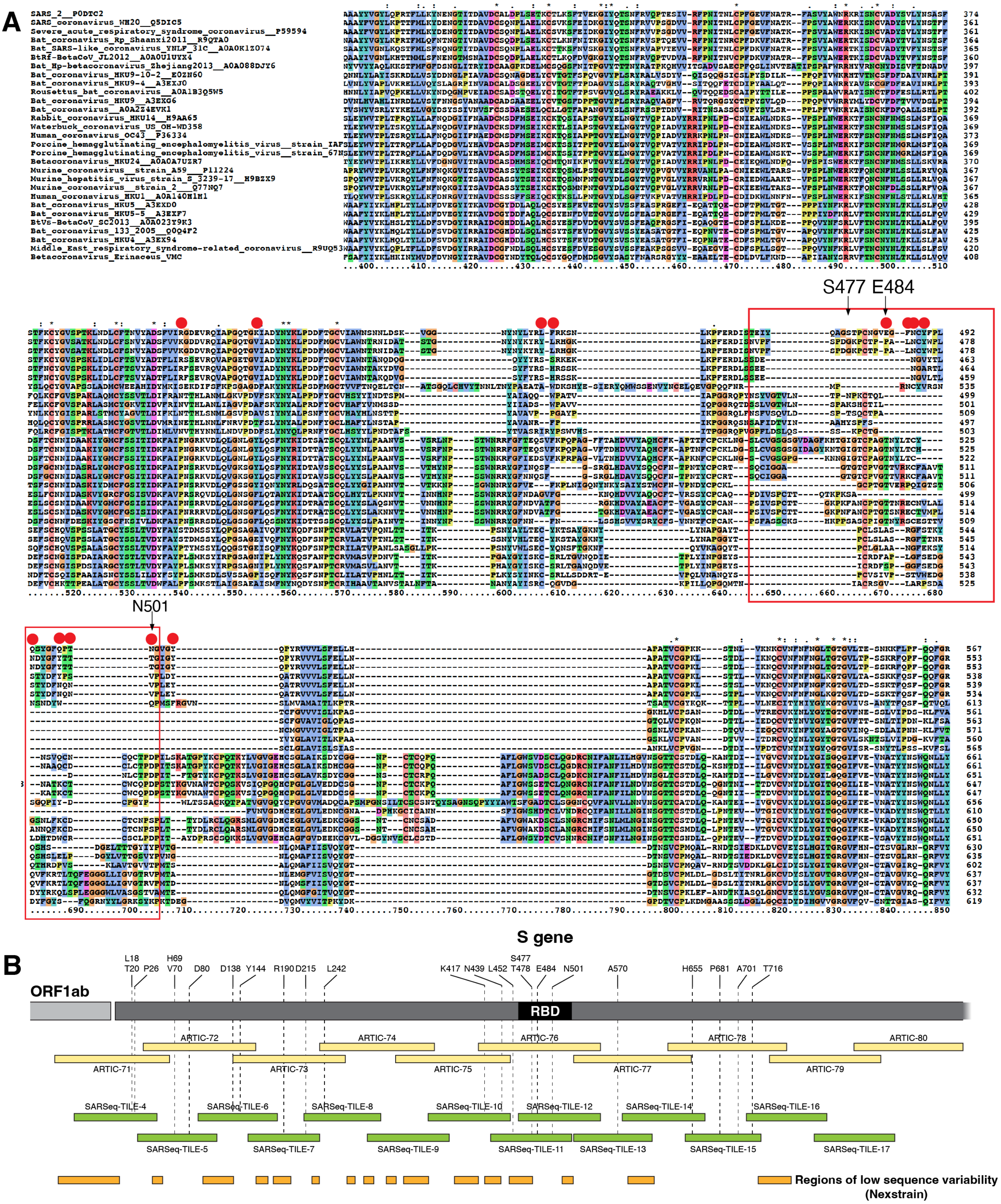

Supplementary Figure S1. Design criteria for the SARSeq S-gene tiles. A. Multiple sequence alignment of the RBD of SARS-CoV-2 (top row) and closely related coronaviruses. The highest degree of variability is seen in the region highlighted by a red frame. Insertions are also seen in some strains in the adjacent protein region. Amino acids interacting with the human ACE2 receptor are highlighted with red circles. Given the importance of this region for neutralizing antibody binding and infectivity it was decided to cover the variable part of the receptor binding domain highlighted with a red frame with two independent amplicons (tiles 11 & 12). **B.** Scheme showing the relative location of ARTIC and SARSeq amplicons in the spike coding sequence. ARTIC amplicons are typically 500 bp long, SARSeq tiles reach a maximum of 281 bp. The region from amino acid 470-502 harboring the important mutations S477N (Omicron), T478K (Delta, Omicron), E484K (Beta, Gamma, E484A in Omicron), N501Y(Alpha, Beta, Gamma, Omicron) is covered with two amplicons in SARSeq. Tiles were designed trying to place primer binding sites in regions of lowest sequence variability (based on Nextstrain data in December 2020).

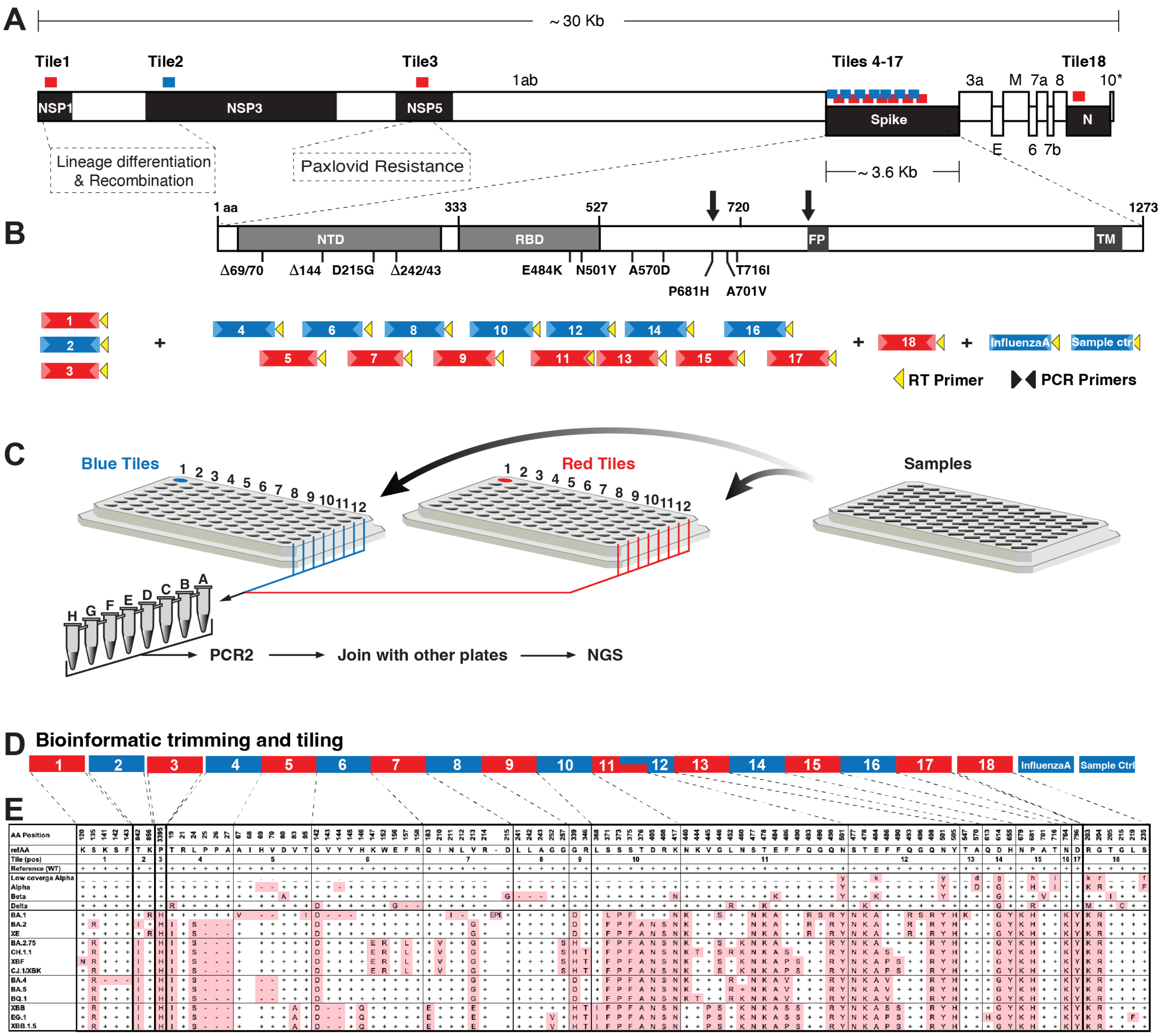

Supplementary Figure S2: SARSeq based pipeline for high throughput sequencing of the SARS-CoV-2 S gene. A. Schematic view of the SARS-CoV-2 genome. **B.** Scheme of the spike protein coding sequence. Known mutations of concern and with phenotypic consequences cluster in the first two thirds of the spike coding sequence including the N-terminal domain (NTD), the receptor binding domain (RBD) up to the protease cleavage sites (arrows) and fusion peptide (FP). Other regions are mostly devoid of such mutations. Tiles to cover the spike coding sequence from amino acids 1-839 are colored blue (Blue tiles) and red (Red tiles), primers used for amplification are highlighted in lighter color. Tile specific RT primers are shown as yellow triangle. During the surveillance period, the pipeline was expanded to include additional amplicons (1, 2, 3, 16, 18, InfluenzaA) beyond the spike protein to differentiate variants, detect recombinants, assess positions associated with potential drug resistance, as well as Influenza A coinfection. Addition of a human RNA-specific amplicon (Sample ctr) ensured monitoring of sample quality. **C.** Blue and Red RT and PCR reactions are performed separately. Plates are subsequently pooled to single columns, where Blue and Red tiles are united, and a single PCR2 reaction is performed for pools of 12 patient samples. All reactions are then combined, gel purified, and sequenced on an Illumina NovaSeq platform. **D.** Overlapping termini of tiles are resected bioinformatically. The intentional overlap between Tiles 11 and 12 over the RBD is reported twice independently. Each fragment is aligned to the consensus sequence and all fragments are stitched together for the final output **E.** The complete sequence is reported as table: reference positions are denoted as "+" or "_" if below confidence cutoff (see text). Amino acid changes are in uppercase for positions with >50% of reads being mutant, but 20%-50% of mutant reads or mutations below cutoff are shown in lowercase. Deletions are denoted by "-" or "." if below cutoff. Blank cells = no reads. Positions 370-502 are reported in duplicate (independent amplicons 11 and 12).

A

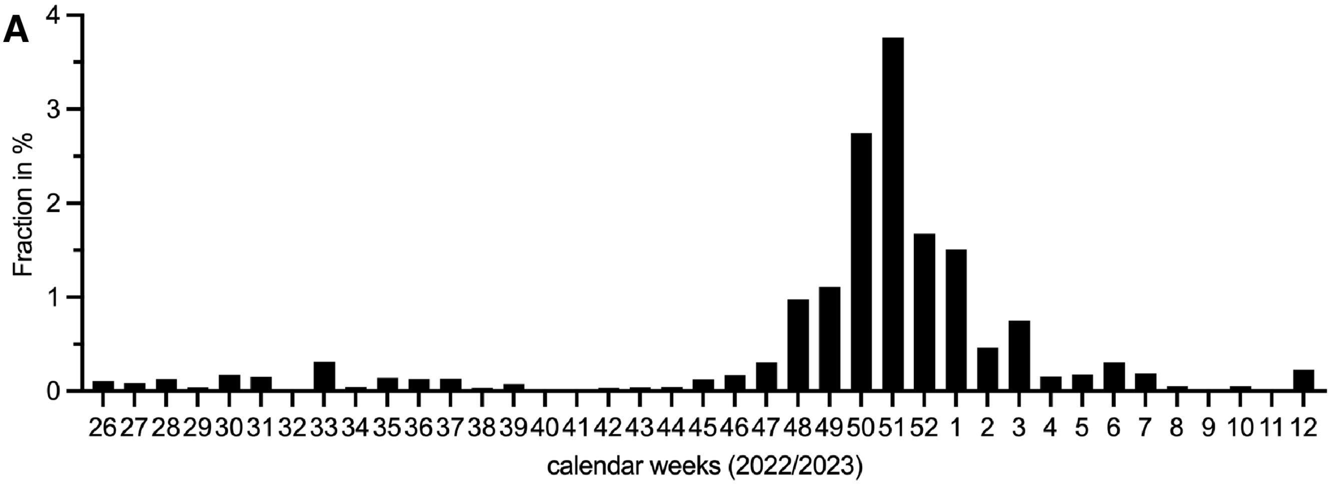

B

posAA	114aa	135aa	842aa	3395aa	19aa	24aa	25aa	26aa	27aa	68aa	69aa	70aa	142aa	153aa	164aa	213aa	245aa	257aa	339aa	371aa	373aa	375aa	376aa	405aa	408aa	417aa
refAA	I	S	T	P	T	L	P	P	A	I	H	V	G	M	N	V	H	G	G	S	S	S	T	D	R	K
tile	1		2	3	4					5			6			7	8		9	10						
Omikron(BA.5)	+	R	I	H	I	-	-	-	-	-	-	-	D	+	+	G	+	+	D	F	P	F	A	N	S	N
Omikron(BA.2.3.20)	+	R	I	H	I	-	-	-	-	+	+	+	D	T	K	G	N	D	D	F	P	F	A	N	S	N
mixed(BA.2.3.20+BA.5)	t	R	I	H	I	-	-	-	-	.	.	.	D	T	K	G	N	D	D	F	P	F	A	N	S	N

posAA	440aa	444aa	450aa	452aa	460aa	477aa	478aa	484aa	486aa	498aa	501aa	477aa	478aa	484aa	486aa	498aa	501aa	505aa	614aa	655aa	679aa	681aa	764aa	796aa	203aa	204aa
refAA	N	K	N	L	N	S	T	E	F	Q	N	S	T	E	F	Q	N	Y	D	H	N	P	N	D	R	G
tile	11											12							14		15		16	17	18	
Omikron(BA.5)	K	+	+	R	+	N	K	A	V	R	Y	N	K	A	V	R	Y	H	G	Y	K	H	K	Y	K	R
Omikron(BA.2.3.20)	K	R	D	M	K	N	K	R	+	R	Y	N	K	R	+	R	Y	H	G	Y	K	H	K	Y	K	R
mixed(BA.2.3.20+BA.5)	K	R	D	r	K	N	K	R	v	R	Y	N	K	R	v	R	Y	H	G	Y	K	H	K	Y	K	R

"+" reference positions
"_" reference positions below confidence cutoff*
"Uppercase amino acid" >50% of reads show a mutation
"Lowercase amino acid" 20%-50% of mutant reads or mutations below cutoff
"-" Deletions
"." Deletions below cutoff
*confidence cutoff = 1% of reads relative to median of the tile and a minimum of 10 reads

Supplementary Figure S3: A. Fraction (in %) of Influenza A in COVID-19 patients per calendar week. **B.** Example of co-infection by two variants BA.5 and BA.2.3.20. Based on the reproducible quantitative assessment of more than one SARS-CoV-2 lineage present in the sample across a wide range on concentrations (**see Supplementary Figure S7**), we also displayed mutations at 20%- 50% of reads in amplicons with good coverage. While mutations present at >50% are displayed as capital letters, mutations present at 20-50% are shown in small letters. This allowed for easy detection and annotation of various cases of mixed genomes due to cross contamination of samples or mixed infection such as the sample with BA.2.3.20 (majority of reads) and BA.5 (minority of reads) with an additional de-novo mutation of I114T.

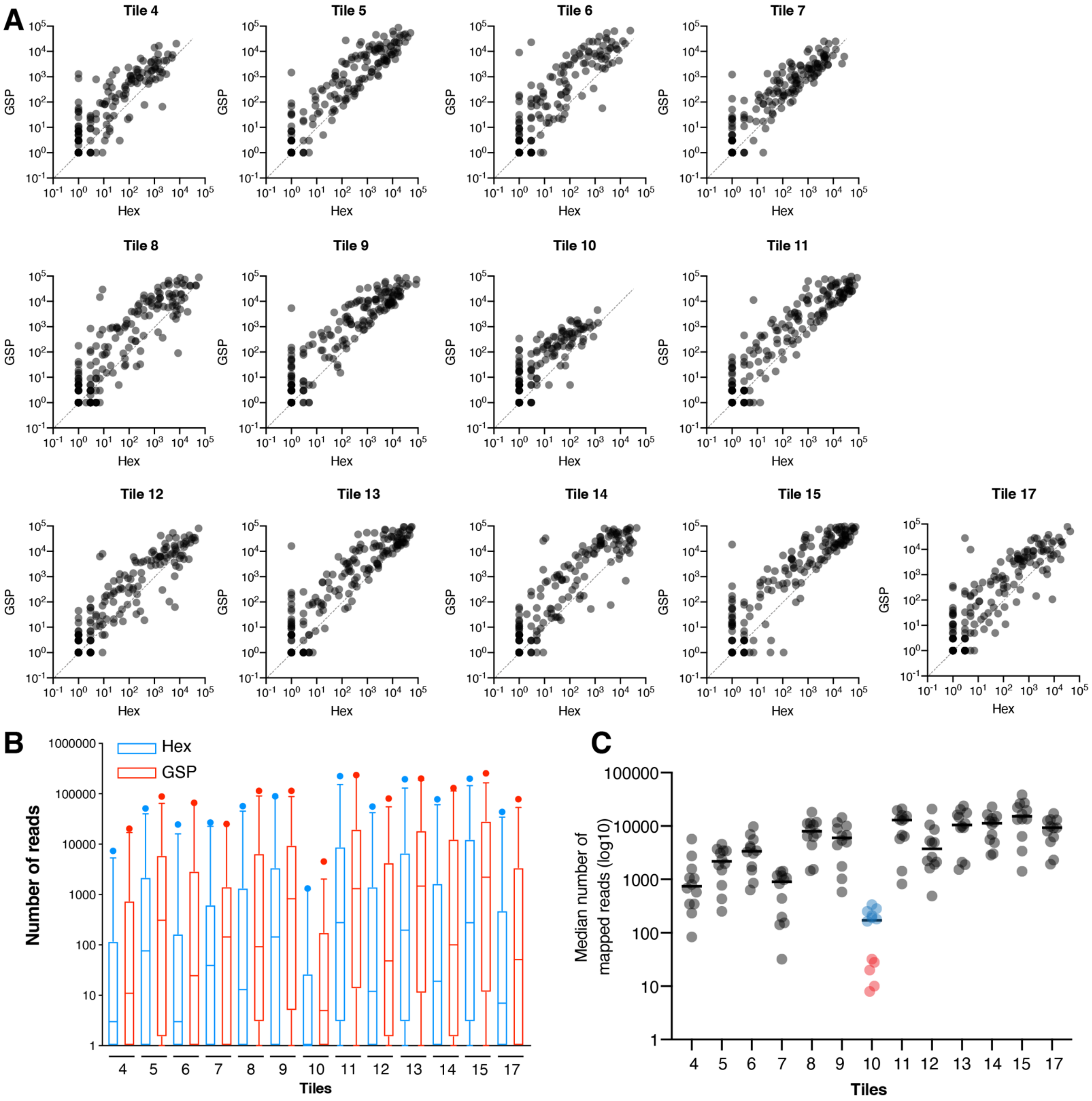

Supplementary Figure S4. Effect of gene-specific priming during RT. A. Scatter plots of read depth obtained by SARSeq S-tiling upon reverse transcription primed either with random hexamers (X-axis) or S-gene specific primers (GSP) with a melting temperature of approximately 58ºC. Each dot represents a clinical sample (n=192). For all tiles, gene specific primers resulted in higher coverage. Note that many samples were only covered with gene specific RT primers, this is particularly evident for amplicon 10 that typically reached lower read depth. **B.** Box plot summarizing data from A. **C.** Median number of reads obtained for each of the 13 tiles, per independent analysis run (each run consisted of ~2,300 samples). Tile 10 reproducibly generates the lowest number of reads, but read number increased about ten-fold when adding a second tile-specific RT primer (blue runs versus red runs).

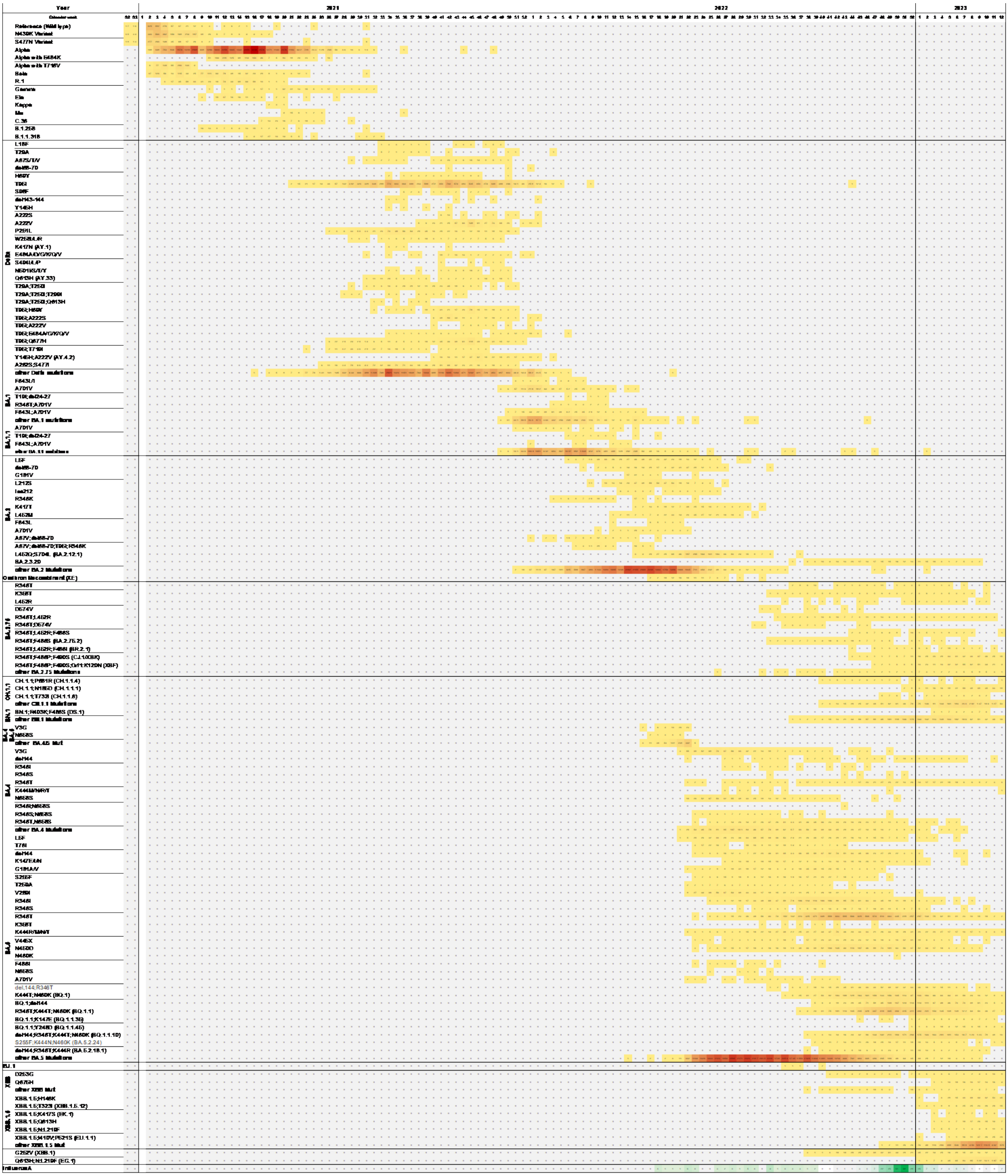

Supplementary Figure S5. Newly emerging single mutations per variant. Numbers of newly emerging single mutations per variant (row) and calendar week (column) (please refer to **Supplementary Table 4 "Mutations", for detailed numerical data**).

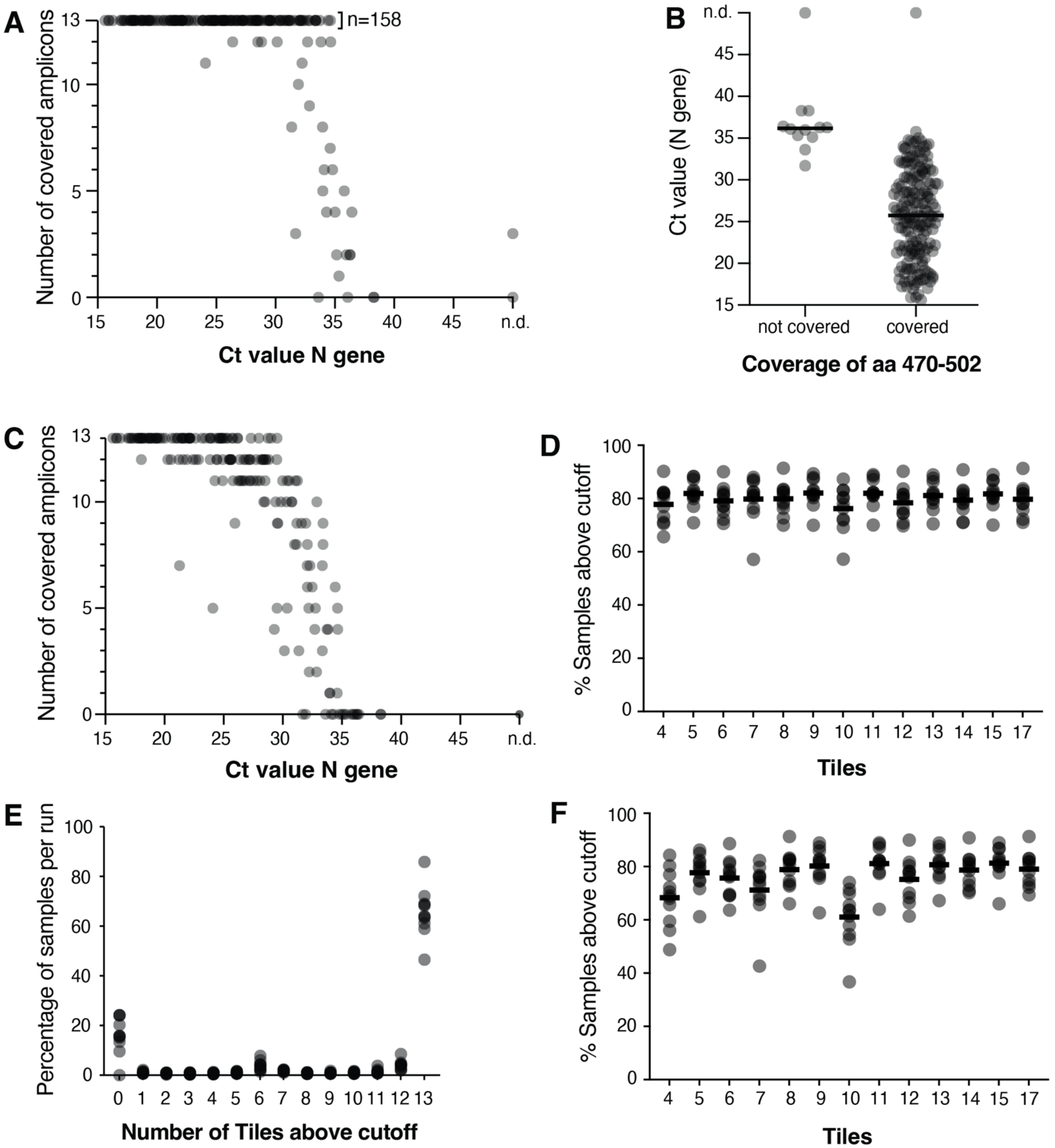

Supplementary Figure S6: **Amplification performance and sensitivity**. **A.** Number of amplicons detected relative to Ct value. Most amplicons are recovered up to Ct~34-35. B. Coverage of the most flexible region of the RBD including most ACE2 interacting positions relative to Ct value. The region is considered covered if Tile 11 or Tile 12 (or both) are detected. **C.** Bioinformatic scaling of recovered amplicons to a hypothetical run of 23,000 samples in a single sequencing lane while maintaining the cutoff rules. Recovery remains near complete at Ct <30. **D.** Percentage of samples with successful detection of each of the 13 tiles above a confidence cutoff of >1% of reads relative to the median reads for that tile obtained in the run (or a minimum of 10 read). Data for 11 independent runs (each with 2,300 samples) is plotted. See Supplementary Table 5 for source data. **E.** Distribution of the number of detected amplicons per sample. Data for the same 11 runs as in D is shown. Samples typically generate all or no amplicons due to the even sensitivity across the covered region. **F**. Bioinformatic scaling of real-life sample data to hypothetical 23,000 samples analyzed on one flow cell while maintaining the cutoff rules. Even under these conditions, most tiles are covered in >70% of samples.

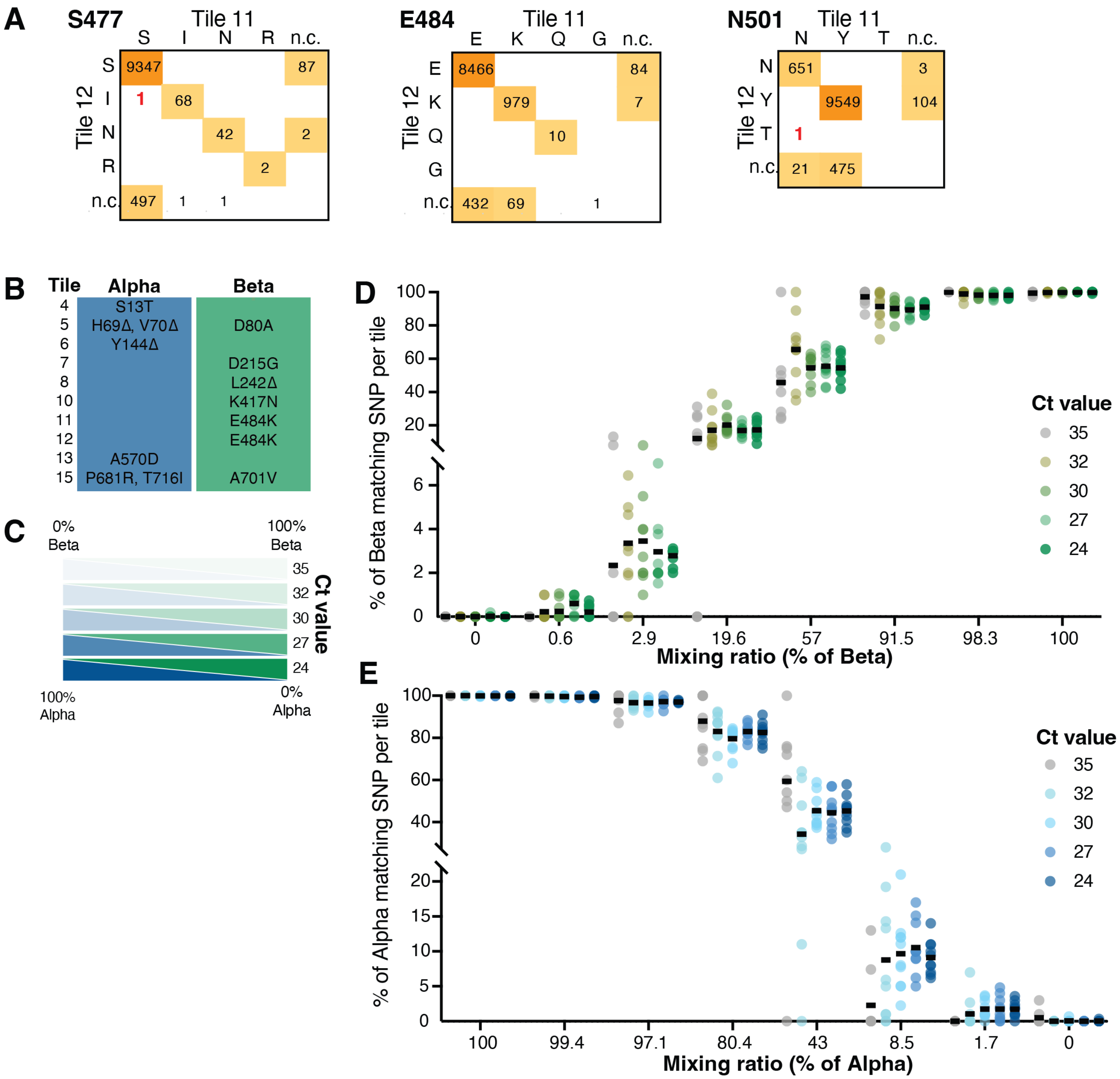

Supplementary Figure S7: Reproducible, sensitive and quantitative detection of different SARS-CoV-2 variants. A. Sequence concordance between amplicons 11 and 12 across >10,000 samples. Columns: results based on amplicons 11, rows: results based on amplicon 12. Two cases of discrepancy within the >30,000 datapoints (samples x positions) are highlighted in red. **B.** Discriminating positions between Alpha with S13T and Beta variants in 10/13 tiles. **C.** Graphical illustration of the experimental outline of mixing two distinct strains at different ratios and a subsequent dilution series. **D.** Fraction of reads indicative of the Beta variant in dependence of Ct values and mixing ratios. Individual datapoints refer to each of the 10 tiles used for the analysis. **E.** Same as in D but for the Alpha variant. As expected, differences across tiles increase with higher Ct values and sub-stochiometric variants are sometimes not detected at high Ct value. See **Supplementary Table 6** for source data.

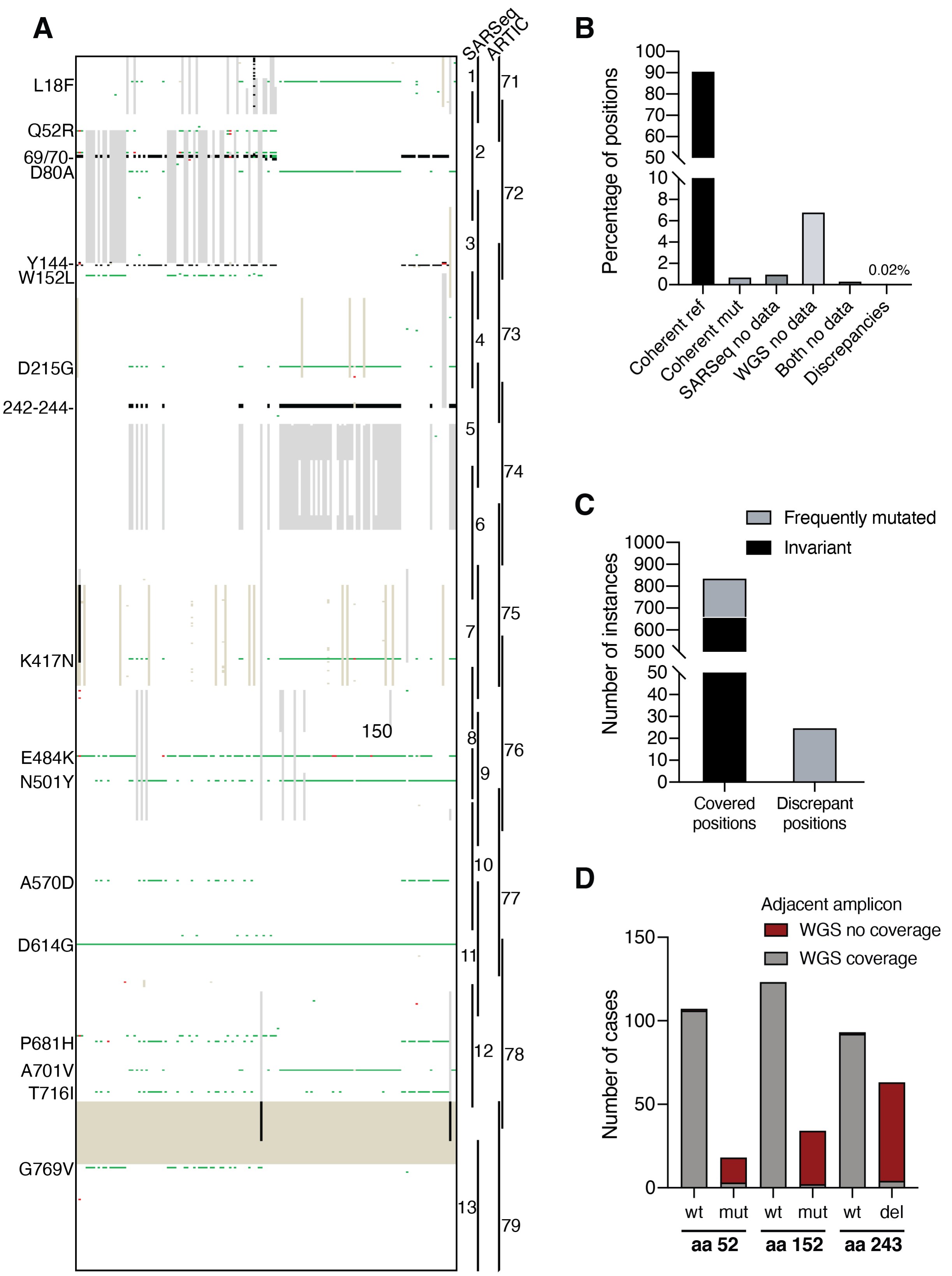

Supplementary Figure S8. Concordance with whole genome sequencing. A. Heat map illustrating agreements and discrepancies between whole genome sequencing using the ARTIC primer set and SARSeq S-tiling based on sequences obtained 2021. Each column is one sample, each row is an amino acid position. White: concordant reference call; green: concordant mutant call; red: discrepant call, gray: sequence missing in WGS; beige: sequence missing in SARSeq. The sequence coverage gap in a preliminary setup of tiles missing tile 16 is indicated as beige block. Black: sequence missing in both datasets including genetic deletions e.g. in positions 242-244. **B.** Quantification of each classification as in A. A total of 30 discrepancies was found accounting for <0.02% of compared positions. **C.** Number of amino acid positions showing change in the analyzed dataset of 30,000 samples or among the 25 discrepant positions where SARSeq calls a mutation (for the other 5 discrepancies, WGS calls a mutation but SARSeq finds the reference nt). Whereas only 179/795 of the analyzed positions show change, all 25 discrepancies occur in variable positions. Mutations are thus non-randomly distributed and therefore not generated by the sequencing pipeline but are a feature of the samples analyzed. **D.** Number of analyzed samples with or without sequence coverage of the amplicon neighboring to mutations in positions 52, 152, and 243. Amplification of neighboring amplicons failed in this version of ARCTIC primers when these mutations are present as they affect the primer binding site.

A

B

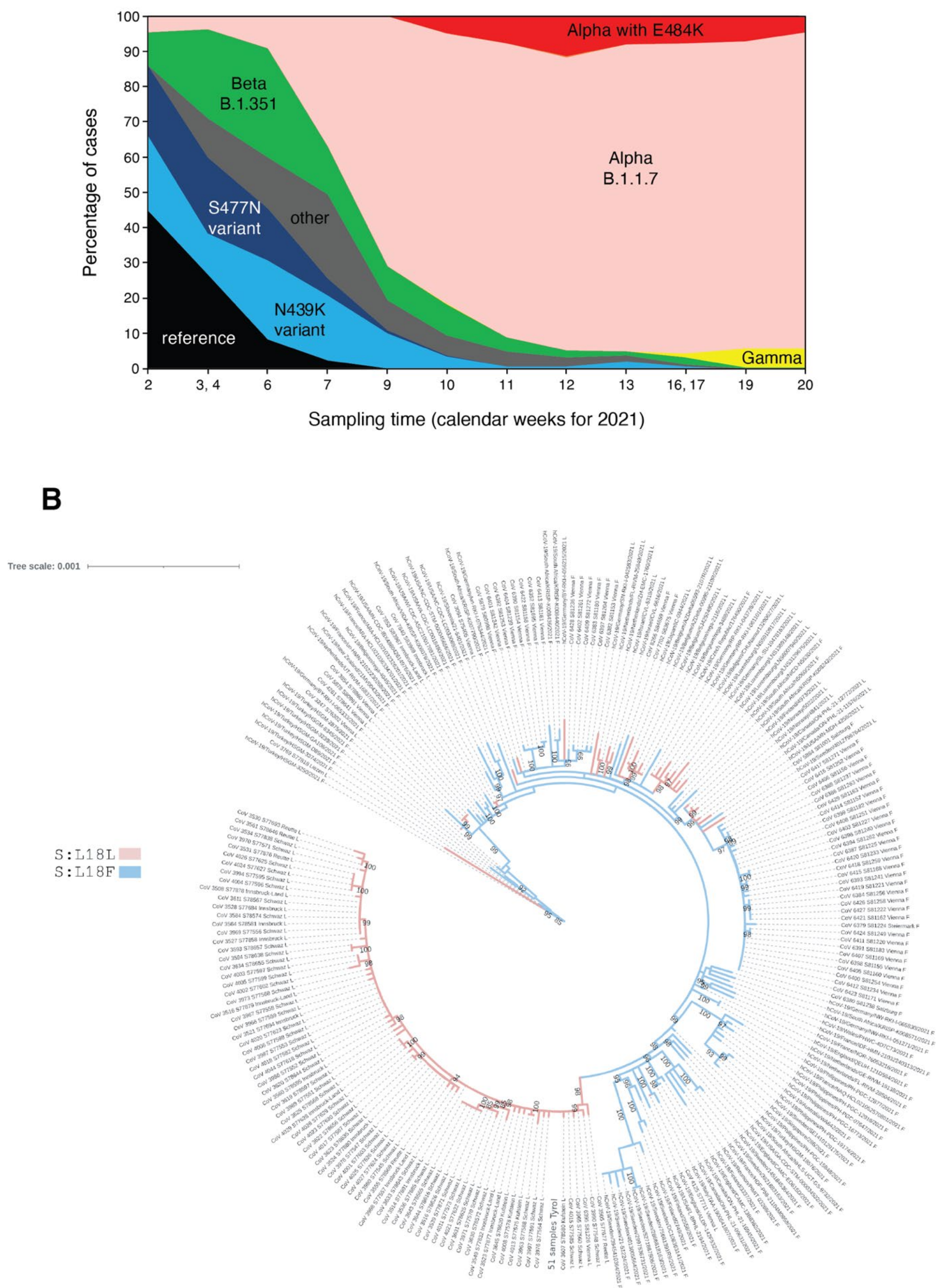

Supplementary Figure S9. Supplementation of SARSeq with WGS. **A.** Timeline of identified strains based on samples analyzed from Tyrol in the period of January to May 2021 identified a cluster of Beta. Results are plotted by calendar-weeks, weeks are joined if individual weeks consisted of <200 datapoints. Total number of samples analyzed is 8,426. Note the early coexistence of Alpha, Beta, other variants, as well as the reference strain. While Beta initially expanded in Tyrol, it was, like all other sub-strains, overtaken by the relative expansion of Alpha. **B.** Whenever relevant, partial genome sequencing of SARSeq was complemented by WGS for cornfirmation of results and to enable phylogenetic analysis. In this example, a phylogenetic tree of Beta (B.1.351) in Austria based on whole genome sequencing ius displayed. International cases are added to put distances in perspective. The position L18 separates the relatively homogenous cluster in Tyrol (L18L, blue) from the majority of cases outside Tyrol (L18F, red). Based on the data in this analysis no spread of the Beta cases of Tyrol to other countries is evident.

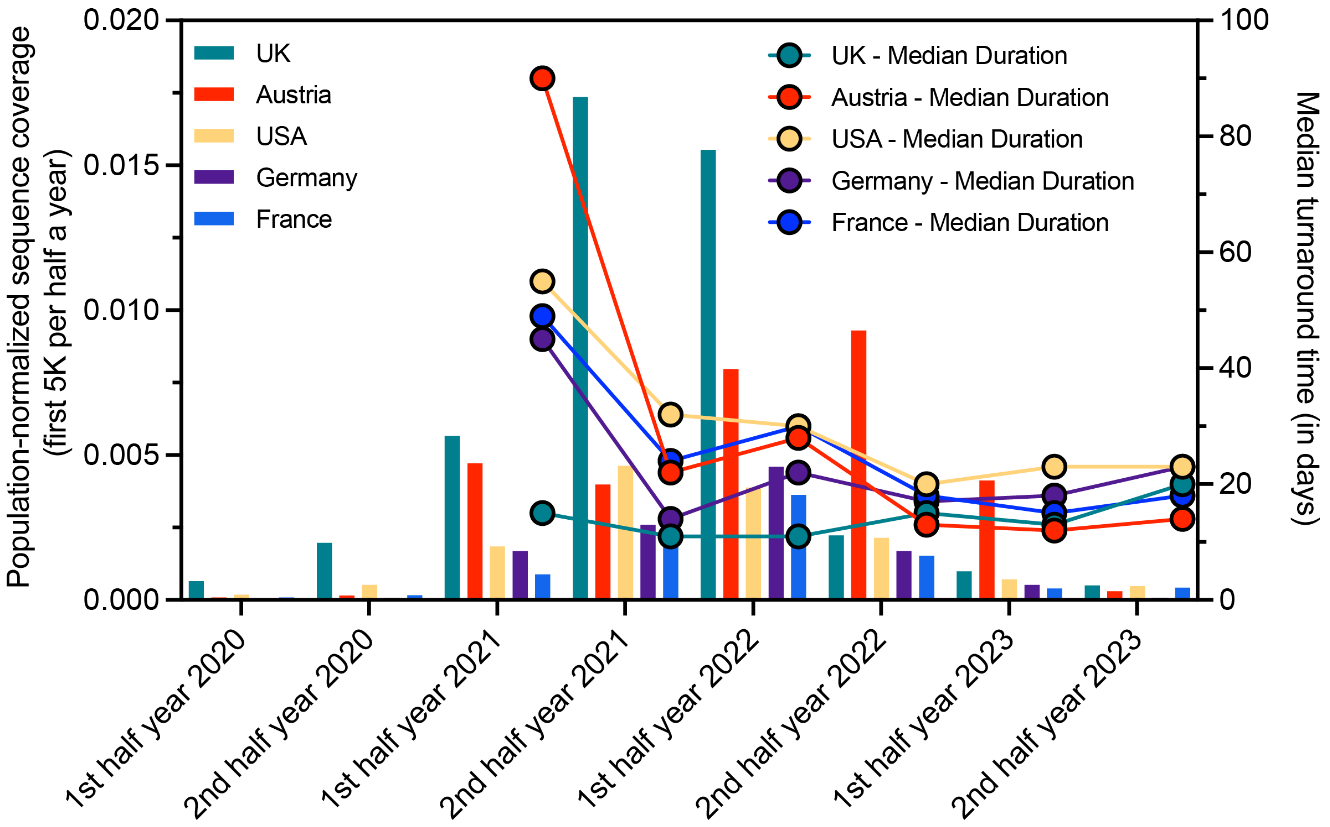

Supplementary Figure S10: Analysis of sequencing density and median turnaround time from several of the largest European countries by population using data from GISAID sequence uploads. Population-normalized sequence coverage was calculated by dividing the total number of sequences from each country per half-year by its population. Median duration was determined by analyzing the first ~5000 sequences from each half-year period. The first half-year period is defined as January 1st to June 30th, and the second half-year period is defined as July 1st to December 31st.

Vienna

CW 51

1	2	3	4	5	6	7	8	9	10	11	12
BF.7	BQ.1.1.10	BF.7	BF.7	BN.1	BF.7	BQ.1.1.10	BA.5	BQ.1.1	BN.1	BN.1	BQ.1.1
XBB/XBB.1	BF.7	BA.5	BF.7	BQ.1.1	XBB.1.5	BF.7	BQ.1.1	BA.5	BQ.1.1	BQ.1.1	BN.1
BQ.1	BF.7	BQ.1	BF.7	BQ.1.1	BF.7	BQ.1	BQ.1.1	BQ.1	BQ.1	BQ.1.1	BF.7
BF.7	XBB.1.5	BF.7	BA.4	BQ.1.1	BQ.1.1.10	BN.1	BA.5	BF.7	BA.2.75	BQ.1.1	BA.5
BQ.1.1.10	XBB/XBB.1	BQ.1.1	BF.7	BF.7	BQ.1.1	BQ.1	BQ.1.1	BQ.1.1.10	BA.5	BQ.1.1	BN.1
BA.5	BF.7	BA.5	BQ.1.1	BA.5	BQ.1	BA.5	BF.7	BQ.1.1	BQ.1.1.10	BQ.1	BA.5
BQ.1.1	BF.7	BF.7	BA.5	BQ.1.1	BF.7	BQ.1.1	BQ.1.1	BA.5	BQ.1.1	BF.7	BQ.1
BQ.1.1	BQ.1.1	BF.7	CH.1.1	BF.7	BN.1	BQ.1.1	CH.1.1	BA.5	BQ.1.1	BF.7	BQ.1

CW 48

1	2	3	4	5	6	7	8	9	10	11	12
BF.7	BA.5	BA.5	BA.5	BF.7	BA.5	BA.5	BF.7	BF.7	BF.7	BQ.1	BF.7
BF.7	BF.7	BF.7	BA.5	BQ.1	BF.7	BA.5	BQ.1.1.10	BN.1	XBB/XBB.1	BQ.1.1	BQ.1.1
BQ.1.1.10	BA.5	BQ.1.1.10	BQ.1.1	BN.1	BQ.1.1.10	BQ.1	BQ.1.1	BA.5	BA.5	BF.7	BA.4
BQ.1	BA.5	BQ.1.1	BA.5	BA.4	BA.5	BA.5	BA.5	BF.7	BF.7	BA.5	
BF.7	BQ.1.1	BA.5	BQ.1.1	BQ.1	BA.5	BA.5	BA.5	BA.5	BQ.1.1	BA.5	BQ.1.1
BQ.1.1	BA.5	BQ.1.1	CH.1.1	BF.7	BF.7	BA.5	BF.7	BF.7	BF.7	BA.5	BQ.1.1
BF.7	BF.7	BA.5	BQ.1.1	BA.5	BN.1	BA.4	BF.7	BA.5	BQ.1.1.10	BA.5	BA.2
BQ.1.1	BA.5	BF.7	BA.5	BA.5	BA.4	BQ.1	BF.7	BQ.1.1	BN.1	BQ.1.1.10	BA.5

Vorarlberg

CW 51

1	2	3	4	5	6	7	8	9	10	11	12
BQ.1.1	BQ.1	CH.1.1	BF.7	BQ.1.1	BF.7	BQ.1.1	BF.7	BQ.1.1	CH.1.1	BQ.1.1	?
BN.1	BQ.1.1.10	CH.1.1	BQ.1	CH.1.1	BF.7	BF.7	CH.1.1	BF.7	CH.1.1	BF.7	CH.1.1
BF.7	BF.7	BQ.1.1		BQ.1.1.10	CH.1.1	CH.1.1	CH.1.1	BA.4	BQ.1.1.10	BA.5	BQ.1
BQ.1	BQ.1.1.10	BQ.1.1	BQ.1.1.10	BQ.1.1.10	BQ.1.1	CH.1.1	CH.1.1	BQ.1.1.10	BQ.1.1	BQ.1.1	BF.7
BA.2.75	BF.7	BF.7	BF.7	BQ.1.1.10	BF.7	BF.7	BQ.1.1	CH.1.1	others	BQ.1	BQ.1.1.10
BA.2.75	BQ.1.1	BQ.1.1	CH.1.1	BQ.1.1	BQ.1.1	BF.7	BQ.1.1.10	BN.1	BQ.1.1.10	CH.1.1	BF.7
BQ.1.1	BQ.1.1.10	CH.1.1	BF.7	BQ.1.1	BF.7	BQ.1	BQ.1.1.10	BQ.1.1.10	BQ.1.1	CH.1.1	BQ.1.1.10
BA.5	BF.7	BQ.1.1	BQ.1.1		BQ.1.1	BQ.1.1.10	CH.1.1	CH.1.1	BQ.1.1	BF.7	BQ.1.1.10

CW 48

1	2	3	4	5	6	7	8	9	10	11	12
BQ.1.1	BQ.1.1	CH.1.1	BQ.1.1	BQ.1.1.10	BF.7	BQ.1	BQ.1.1	BQ.1.1	BA.5	BA.5	BQ.1.1
BQ.1.1	CH.1.1	BQ.1.1	BA.2.75	BF.7	BF.7	BA.5	BQ.1	BQ.1	BQ.1.1	BQ.1.1.10	BF.7
BF.7	BA.5	BA.4	BQ.1.1	BA.5	BF.7	BF.7	BA.2.75	BQ.1	BF.7	BQ.1.1.10	BQ.1.1
BQ.1.1.10	CH.1.1	BQ.1.1	BQ.1	BQ.1	BA.5	BQ.1	BF.7	BA.5	BQ.1.1.10	BA.5	BQ.1.1.10
BQ.1.1	BQ.1.1.10	BF.7	BF.7	BQ.1.1.10	BA.2.75	BQ.1.1.10	BF.7	BQ.1.1.10	BQ.1.1	BN.1	BQ.1.1
BA.2.75	BF.7	BA.5	BQ.1.1.10	BA.5	BF.7	BF.7	BQ.1.1.10	BF.7	BF.7	BQ.1.1.10	BQ.1.1
CH.1.1	BQ.1.1	BF.7	BA.5	BQ.1.1	BF.7	BA.4	BN.1	BF.7	BQ.1	BQ.1.1.10	BF.7
BQ.1.1	BF.7	BQ.1.1	BF.7	BA.5	BQ.1	BQ.1.1.10	BF.7	BQ.1.1	BA.5	BQ.1.1	BQ.1.1

Salzburg

CW 51

1	2	3	4	5	6	7	8	9	10	11	12
BQ.1.1	BQ.1.1	BA.5	BQ.1.1	BA.4	BF.7	BQ.1.1	BN.1	BQ.1.1.10	others	BQ.1	BQ.1.1
BQ.1.1	BQ.1.1.10	BQ.1.1	BQ.1.1.10	BA.5	BN.1	XBB/XBB.1	BQ.1.1		BN.1	BN.1	BF.7
BQ.1.1	BA.5	BQ.1	BA.5	BN.1	BA.5	BF.7	BQ.1	BQ.1.1.10	BQ.1.1	BF.7	BN.1
BQ.1	CH.1.1	BQ.1.1.10	BQ.1.1	BQ.1.1	BA.5	XBB.1.5	BN.1	BN.1	XBB/XBB.1	CH.1.1	BQ.1.1
BQ.1	BQ.1.1	BQ.1.1	BN.1	BF.7	XBB/XBB.1	BN.1	BA.5	BQ.1.1.10	BQ.1	BQ.1.1.10	BQ.1.1
BN.1	BF.7	BF.7	BA.5	BQ.1.1	BN.1	BF.7	CH.1.1	BQ.1.1.10	BF.7	CH.1.1	XBB/XBB.1
BF.7	BA.5	BQ.1	BQ.1.1	BF.7	others	BQ.1.1.10	BQ.1.1	BQ.1.1	CH.1.1	BQ.1	
BN.1	BQ.1	BA.5	BQ.1.1.10	others	XBB/XBB.1	BN.1	BA.5	BQ.1.1.10	BA.5		

CW 48

1	2	3	4	5	6	7	8	9	10	11	12
XBB/XBB.1	BQ.1.1.10		BF.7	BQ.1	BF.7	BA.5	BA.5	BA.5	BF.7	BA.5	BF.7
BA.5	BQ.1.1	BA.5	BA.5	BA.5	BA.5	BA.5	CH.1.1	BA.5	BF.7	BF.7	BQ.1
BQ.1.1	BQ.1.1	BQ.1.1.10	BN.1	BF.7	BQ.1.1	BA.5	BA.5	BQ.1	CH.1.1		BA.5
BQ.1	BQ.1.1.10	BA.5	BA.4	BA.4	BA.5	BA.2.75	BF.7	BN.1	BA.5	BF.7	CH.1.1
BF.7	BF.7	BA.5	BA.2.75	BA.5	BF.7	BQ.1	BA.5	BF.7	BQ.1.1	BQ.1.1	BA.5
BF.7	BA.5	BA.5	BA.5	BA.5	BF.7	BA.5	BQ.1.1	BQ.1	BQ.1.1	BA.5	BF.7
BQ.1	BA.5	BA.5	BF.7	BA.5	BQ.1	CH.1.1	BA.5	BQ.1.1	BA.5	BA.5	?
XBB/XBB.1	BF.7	BQ.1.1.10	BA.5	BA.5	BQ.1.1	BF.7	BQ.1.1	BA.5	BA.5		?

Styria

CW 51

1	2	3	4	5	6	7	8	9	10	11	12
BQ.1.1	BQ.1.1	BQ.1.1	BF.7	BA.5	BQ.1	BQ.1.1	BQ.1.1	BA.5	BA.5	CH.1.1	BQ.1
BF.7	BQ.1	BQ.1	BF.7	BQ.1	BA.5	BQ.1	BQ.1.1	BF.7	BQ.1.1.10	BA.5	BQ.1
BQ.1.1.10	BA.5	BQ.1.1.10	BQ.1.1	BQ.1	BA.5	BA.5	XBB.1.5	BA.5	BQ.1	BA.5	BF.7
CH.1.1	BQ.1	BQ.1.1	BF.7	BQ.1.1	BQ.1.1	BQ.1.1	BF.7	BQ.1	BQ.1	BN.1	BQ.1.1
BQ.1	BA.5	BF.7	BQ.1.1	CH.1.1	BA.5	BA.4	BF.7	BA.5	BQ.1.1	XBB.1.5	BA.4
BF.7	BF.7	XBB.1.5	BQ.1.1	BA.5	BA.5	BQ.1.1	BQ.1.1.10	BQ.1.1	BQ.1.1	BA.2.75	BQ.1
BQ.1.1	BA.5	BQ.1.1	BQ.1.1	BA.5	BQ.1.1.10	BA.2.75	BA.5	BQ.1.1	BQ.1.1	BQ.1.1	BA.5
BA.5	BQ.1.1	BA.5	BF.7	BQ.1.1	BQ.1	BF.7	BF.7	BA.4	BQ.1.1	BA.5	BQ.1

CW 48

1	2	3	4	5	6	7	8	9	10	11	12
BA.5	BN.1	BF.7	BA.5	BA.5	BA.5	BQ.1	BA.2.75	BQ.1	BQ.1	BQ.1.1	BF.7
BQ.1.1	BF.7	BQ.1	BF.7	BA.5	BA.5	BA.5	BA.5	BA.4	BQ.1.1	BA.5	BQ.1.1
BF.7	BF.7	BA.5	BQ.1.1.10	BA.5	BA.5	BQ.1.1	BF.7	BA.5	BQ.1.1	BA.5	others
BQ.1.1.10	BF.7	BQ.1.1	BA.5	BA.5	BA.5	BN.1	BA.5	BA.5	BA.5	BA.5	BA.5
BA.5	BA.5	BF.7	BA.4	BQ.1	BQ.1.1	BA.5	BA.5	XBB/XBB.1	BQ.1.1	BA.4	BA.5
BA.2.75	BA.5	BQ.1	BF.7	XBB/XBB.1	BQ.1.1	BA.5	BQ.1.1	BA.5	BA.5	BA.5	BA.5
BA.5	BQ.1	BA.5	BQ.1.1	BQ.1.1.10	BF.7	BQ.1	BQ.1.1	BF.7	BA.5	BF.7	BQ.1.1
BQ.1.1	BA.5	BQ.1	BA.5	BA.5	BQ.1.1	BQ.1	BF.7	BA.2.75	BQ.1.1	BQ.1	BQ.1.1

Tyrol

CW 51

1	2	3	4	5	6	7	8	9	10	11	12
		BQ.1.1	BQ.1	BF.7	XBB/XBB.1	BQ.1	BQ.1.1	BA.2.75	BQ.1	BA.4	BQ.1.1.10
BN.1	BQ.1	BF.7	BQ.1.1.10	BQ.1.1	BQ.1.1	BQ.1.1.10	BF.7	BQ.1.1.10	BQ.1	CH.1.1	BQ.1
BQ.1.1	BA.4	BQ.1	BQ.1.1	BQ.1.1	BQ.1.1.10	BQ.1	BF.7	others	BF.7	BQ.1	
BF.7	BF.7	BF.7	BQ.1	BA.5	BQ.1.1	BQ.1	BQ.1.1	BF.7	BQ.1.1	BF.7	BQ.1.1.10
BF.7	BQ.1	BQ.1.1	BQ.1	BQ.1.1.10	BQ.1.1	BQ.1	BQ.1.1	BQ.1.1	BF.7	BQ.1.1.10	BQ.1.1
BN.1	XBB/XBB.1	BN.1	BQ.1.1.10	XBB/XBB.1	BQ.1.1	BQ.1.1	BN.1	BQ.1	BQ.1.1	BQ.1	BF.7
BQ.1	BQ.1.1	BQ.1.1	BQ.1.1	CH.1.1	BQ.1.1	BF.7	BF.7	BQ.1.1	BQ.1.1.10	BQ.1	XBB/XBB.1
BQ.1.1	BQ.1.1	BQ.1.1	BN.1	BQ.1	XBB/XBB.1	BQ.1.1	BQ.1	BF.7	BN.1	BQ.1.1	BQ.1.1

CW 48

1	2	3	4	5	6	7	8	9	10	11	12
		BA.5	BQ.1	BF.7	BQ.1	XBB/XBB.1	BQ.1	BQ.1	BA.5	BA.5	BA.5
BF.7	BA.5	BQ.1.1	BQ.1.1	BQ.1	BA.5	BF.7	BQ.1	BA.5	BF.7	BQ.1.1	BA.5
BQ.1.1.10	BF.7	BQ.1.1	BQ.1.1	BA.5	BQ.1	BF.7	BQ.1	BQ.1	BQ.1	BQ.1.1.10	BQ.1.1
BQ.1.1	BA.5	BF.7	BQ.1	BA.5	BF.7	BA.5	BF.7	BQ.1.1	BQ.1	BQ.1.1.10	BA.5
BA.5	BQ.1.1	BF.7	BQ.1.1	BQ.1.1.10	BQ.1.1.10	BF.7	BQ.1.1	BF.7	BQ.1	BQ.1.1.10	BQ.1.1.10
BF.7	BA.5	BA.5	XBB/XBB.1	others	BQ.1.1	BQ.1.1	BQ.1.1	BF.7	BF.7	BA.5	BF.7
BQ.1.1	BQ.1.1	XBB/XBB.1	BQ.1.1.10	BN.1	BQ.1.1	BQ.1.1	BQ.1	BQ.1.1.10	BQ.1	BQ.1.1	BQ.1.1.10
BA.5	BA.5	BQ.1	BQ.1.1.10	BQ.1.1	XBB/XBB.1	BA.4	BQ.1.1.10	BQ.1	BQ.1.1.10	BA.5	BF.7

Lower Austria

CW 51

1	2	3	4	5	6	7	8	9	10	11	12
BQ.1	BQ.1	BQ.1	BA.5	BQ.1.1	XBB/XBB.1	BF.7	BF.7	BN.1	XBB/XBB.1	BQ.1	XBB.1.5
BF.7	BQ.1	BQ.1.1	BQ.1.1	BQ.1.1	BF.7	BN.1	BF.7	BQ.1.1	BQ.1	BA.5	BQ.1.1
BF.7	XBB/XBB.1	BF.7	BA.5	BQ.1		BQ.1	BA.5	BQ.1.1.10	BQ.1.1	BA.4	BQ.1.1
BA.5	BN.1	BF.7	BQ.1.1	BQ.1	BF.7	BQ.1.1.10	BN.1	BF.7	BQ.1	BQ.1	BN.1
BA.5	CH.1.1	BF.7	BF.7	BQ.1.1	BQ.1	BF.7	BQ.1	BQ.1	BA.5	BA.5	BF.7
BA.2.75	BN.1	BF.7	BA.5	BQ.1.1	BF.7	BQ.1.1	BQ.1	BF.7	BN.1	BF.7	BF.7
BF.7	BA.5	BQ.1	CH.1.1	BQ.1.1	BQ.1.1	BF.7	BF.7	BA.5	BA.5	BQ.1	BQ.1.1.10
BN.1	BF.7	BF.7	BA.5	BQ.1.1	BF.7	BF.7	BF.7	BA.5	BF.7	BA.5	BQ.1

CW 48

1	2	3	4	5	6	7	8	9	10	11	12
BF.7		BA.5	BQ.1.1	BA.5	BA.5	BA.5	BA.5	BQ.1.1	CH.1.1	BF.7	BF.7
BF.7	BQ.1.1	BA.5	BQ.1.1	BA.5	BA.5	BF.7	BA.5	BF.7	BA.5	BQ.1.1.10	BA.5
BQ.1.1	BA.5	BQ.1.1	BA.5	BA.5	BF.7	CH.1.1	BA.4	BQ.1.1	BA.5	BQ.1.1	BF.7
BQ.1.1		BQ.1	BF.7	BA.5	BF.7	BF.7	BF.7	BA.5	BF.7	BF.7	BA.5
BF.7	BA.5	BA.5	BF.7	BQ.1.1.10	BA.4	BN.1	BF.7	BN.1	BQ.1.1	BQ.1	BF.7
BF.7	BF.7	BA.5	BA.5	XBB/XBB.1	BQ.1.1	BF.7	BF.7	BQ.1.1	BQ.1.1.10	BQ.1.1	BF.7
BF.7	BA.5	BA.5	BF.7	BF.7	BF.7	BA.5	BA.5	BA.5	BQ.1.1	BA.5	BF.7
BA.5	BA.5	BQ.1	BF.7	BF.7	XBB/XBB.1	BQ.1.1	BQ.1.1	BQ.1.1	BA.5	BF.7	BF.7

Carinthia

CW 51

1	2	3	4	5	6	7	8	9	10	11	12

CW 48

1	2	3	4	5	6	7	8	9	10	11	12
BA.5	BA.5	BF.7	BQ.1	BA.5	BA.5	BA.5	BF.7	BF.7	BF.7	BQ.1.1	BQ.1.1
BQ.1.1	BF.7	BA.5	BQ.1.1	BA.5	BA.5	XBB/XBB.1	BA.5	BA.5	BF.7	BA.5	BA.5
BA.5	BF.7	BA.2.75	BF.7	BA.5	BA.5	BF.7	BA.4	BN.1	BF.7	XBB/XBB.1	BA.4
BF.7	BF.7	BQ.1.1	BQ.1.1	BA.5	BQ.1	BF.7	BF.7	BA.5	BF.7	BQ.1	BA.5
BN.1	BA.5	BF.7	BA.5	BF.7	BF.7	BF.7	BN.1	BF.7		XBB/XBB.1	BQ.1.1
BA.5	BA.5	BA.2.75	BF.7	BA.5	BA.5	BA.5	BA.5	BQ.1	BA.5	BA.4	BF.7
BQ.1.1	BA.5	BF.7	BA.5	BF.7	BA.5	BF.7	BF.7	BQ.1		BA.5	BA.5
BA.5	BF.7	BA.5	BF.7	BQ.1	BQ.1	others	BA.5	BF.7	BA.5	BF.7	BF.7

Upper Austria

CW 51

1	2	3	4	5	6	7	8	9	10	11	12
BA.5	BQ.1	BF.7	BF.7	BQ.1.1							
BA.5	BA.5	BA.5	BQ.1	BA.5							
BA.5	BQ.1	BA.5	BA.5	BQ.1							
BQ.1	BQ.1	BA.5	XBB/XBB.1	BA.5							
BA.5	BF.7	BA.5	BF.7	BA.5							
BA.5	BQ.1.1	BQ.1.1.10	BQ.1	BF.7							
BA.5	BQ.1	BF.7	BF.7								
BQ.1.1.10	BQ.1.1	BA.5	BQ.1								

CW 48

1	2	3	4	5	6	7	8	9	10	11	12
			BA.4	BA.5	BQ.1.1	BA.5	BN.1				
			BF.7	BA.5	BQ.1.1	BA.5					
			BA.2.75	BA.5	BA.5	XBB/XBB.1	BA.5				
			BA.5	BA.5	BQ.1	BQ.1.1	BQ.1.1				
			BA.5	BF.7	BN.1	BA.5					
			BA.5	BA.5	BA.5	BQ.1					
			BN.1	BQ.1.1.10	BA.5	BA.5					
		BQ.1.1	BA.5	BF.7	BA.5	BQ.1.1					

Burgenland

CW 51

1	2	3	4	5	6	7	8	9	10	11	12
BA.5	BF.7	BA.5	BQ.1.1	XBB/XBB.1	BQ.1.1	BA.5	BQ.1.1.10	BQ.1.1	BF.7	BA.5	BA.5
BA.5	BQ.1.1.10	BA.5	BF.7	BA.5	BA.5	BF.7	BN.1	BQ.1.1	BQ.1	BQ.1	BA.5
BA.5	BF.7	BF.7	BQ.1.1	BF.7	BQ.1.1	BF.7	BA.5	BQ.1.1	BF.7	BF.7	BA.5
BA.5	BQ.1.1	BF.7	BA.5	BF.7	BQ.1.1	BQ.1.1	BF.7	BQ.1.1	BA.5	BQ.1.1	BA.5
BQ.1.1	XBB/XBB.1	BA.5	BF.7	BA.4	BQ.1.1.10	BF.7	BF.7	BA.4	BA.5	BQ.1.1.10	BF.7
BA.5	BQ.1.1.10	BA.5	BA.5	BA.4	BA.5	BF.7	BF.7	BF.7	BF.7	BA.5	BQ.1.1.10
BA.5	BA.5	BA.5	BN.1	BA.5	BQ.1.1	BQ.1	BA.5	BF.7	BQ.1.1.10	BQ.1.1.10	BA.5
BA.5	BA.5	BA.5	BA.5	BQ.1.1	CH.1.1	BQ.1.1.10	BQ.1.1	BF.7	BA.5	BQ.1.1.10	BF.7

CW 48

1	2	3	4	5	6	7	8	9	10	11	12
BQ.1	BF.7	BA.5	BA.5	BA.5	BA.2.75	BA.5	BA.5	BA.5	BF.7	BA.5	BA.5
BA.2.75	BA.5	BQ.1.1	BA.5	BQ.1.1	BA.2.75	BA.4	BF.7	BA.5	BA.5	BA.5	BF.7
BA.5	BA.4		BA.5	BA.5	BA.2.75	BQ.1	BA.5	BA.5	BF.7	BQ.1	XBB/XBB.1
BA.5	BA.5	BF.7	CH.1.1	BA.5	BQ.1.1	BA.5	BF.7	others	BA.5	BF.7	BF.7
BA.5	BF.7	others	BA.4	BA.5	BF.7	BF.7	BN.1	BF.7	BQ.1.1	BA.5	BF.7
BA.5	BA.5	BN.1	BQ.1.1	BA.5	BA.5	BF.7	BQ.1.1	BF.7	BQ.1.1	BF.7	BA.4
BF.7	BA.5	BA.5	BA.5	BA.5	BA.5	BF.7	BA.5	BA.2.75	BQ.1	BF.7	BA.5
BA.5	BQ.1	BA.5	BA.5	XBB/XBB.1	BA.5	BF.7	BQ.1.1	BQ.1.1	BA.5	XBB/XBB.1	BQ.1.1

Supplementary Figure S11: Cluster investigation based on sample distribution per plate for each province. This analysis was conducted with samples from the period when several variants co-existed (mixed variants) (calendar weeks 48 and 51 of 2022).

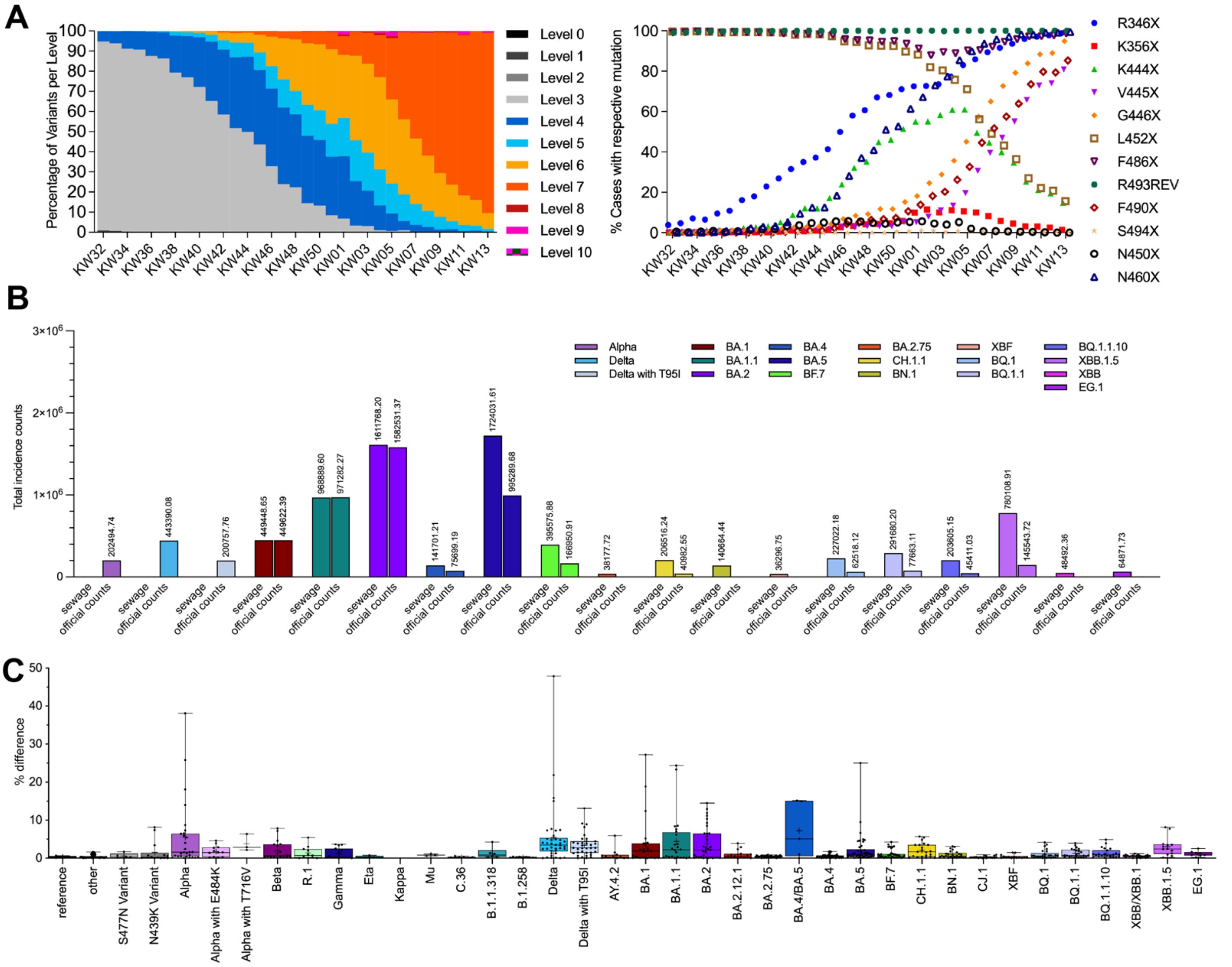

Supplementary Figure S12: Analysis of spike protein mutations, variant infection trends, and share predictions A. Analysis of the most significant mutations under positive selection relative to BA.2 in spike protein (convergent evolution). Left: Overview of relevant variants per analysis week based on the number of additional mutations (Level 0: BA.2, Level 3: e.g., BA.5). Right: relative abundance of each of the twelve significant mutations per analysis week. **B.** Total number of detected infections by different variants (only variants with >5000 cases are shown), based on officially reported case counts (official counts) and on sewage monitoring data (sewage) (**Supplementary Table 4, Incidence counts)**. **C.** Difference of the predicted variant share to the original in absolute numbers.

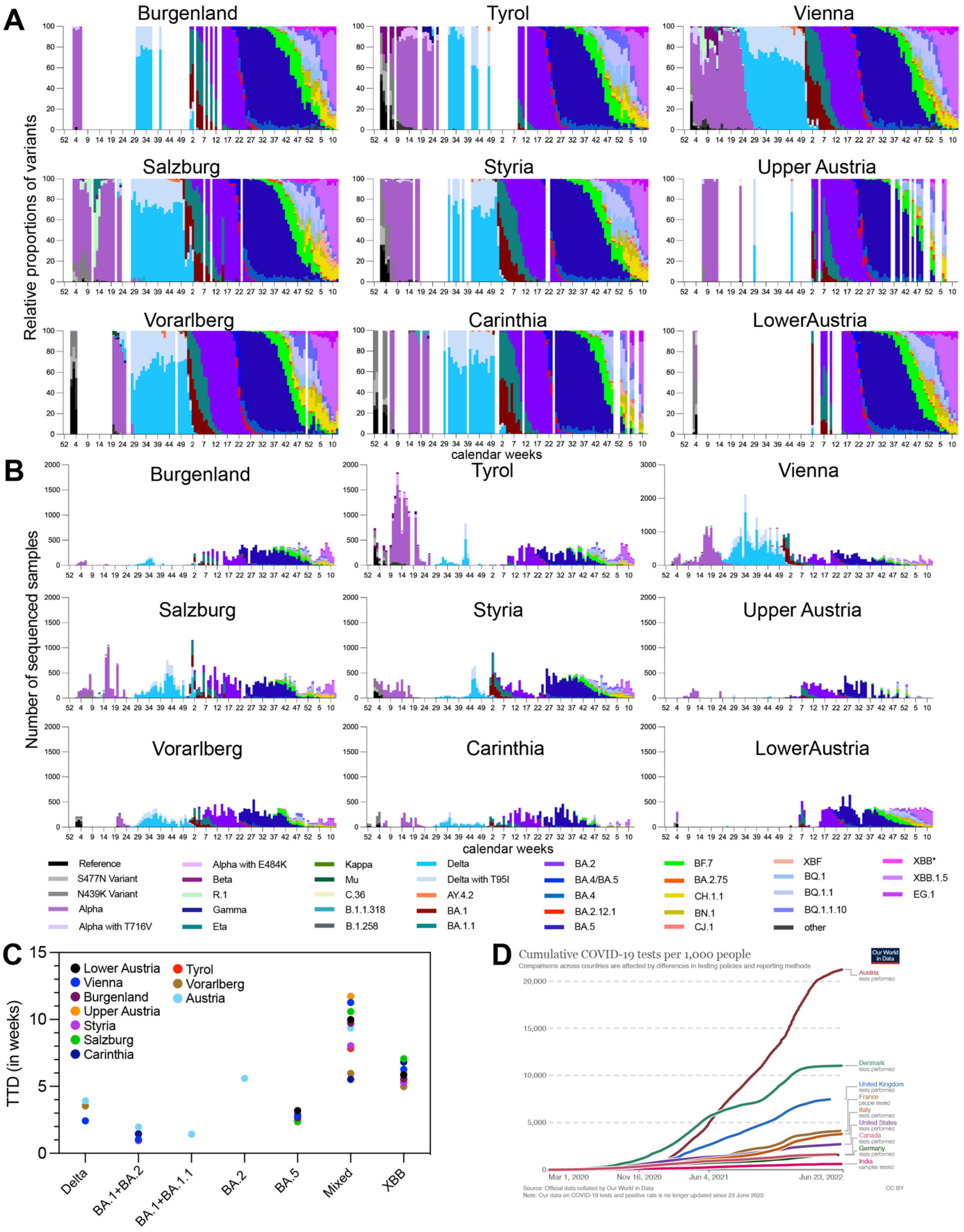

Supplementary Figure S13: **Insights into variant-dynamics, sequencing coverage and time to dominance at province resolution, and testing rates among different states. A.** Timelines of relative proportions of variants per calendar week across all nine Austrian provinces. **B.** Timelines of successfully sequenced samples per calendar week across Austrian provinces. **C.** Time to Dominance (TTD) in weeks for major variant groups (except Alpha) in Austrian provinces and Austria as a whole. TTD is defined as the time period from 10% to 60% prevalence using fitted curves (**Supplementary Figure 12**) **D.** Cumulative COVID-19 tests per 1000 people for Austria compared to other states (Source: Our World in Data).

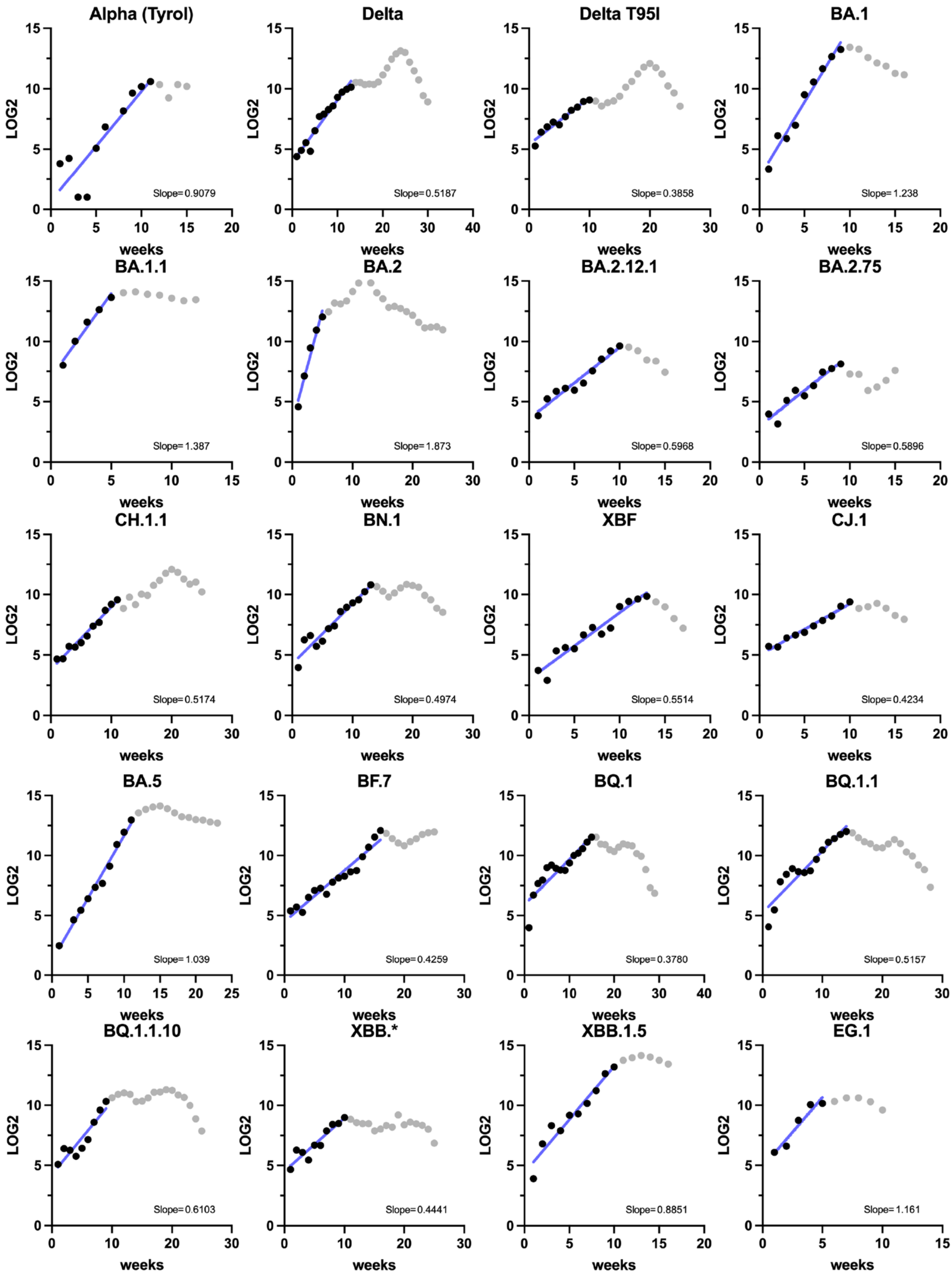

Supplementary Figure S14: **Total incidence numbers in LOG2 plotted per diagnostic week for each individual variant.** The doubling time for each variant was calculated as the reciprocal of the slope of the fitted curve (blue line) (for **Figure 3B**, refer to **Supplementary Table 4, "doubling time",** for detailed numerical data).

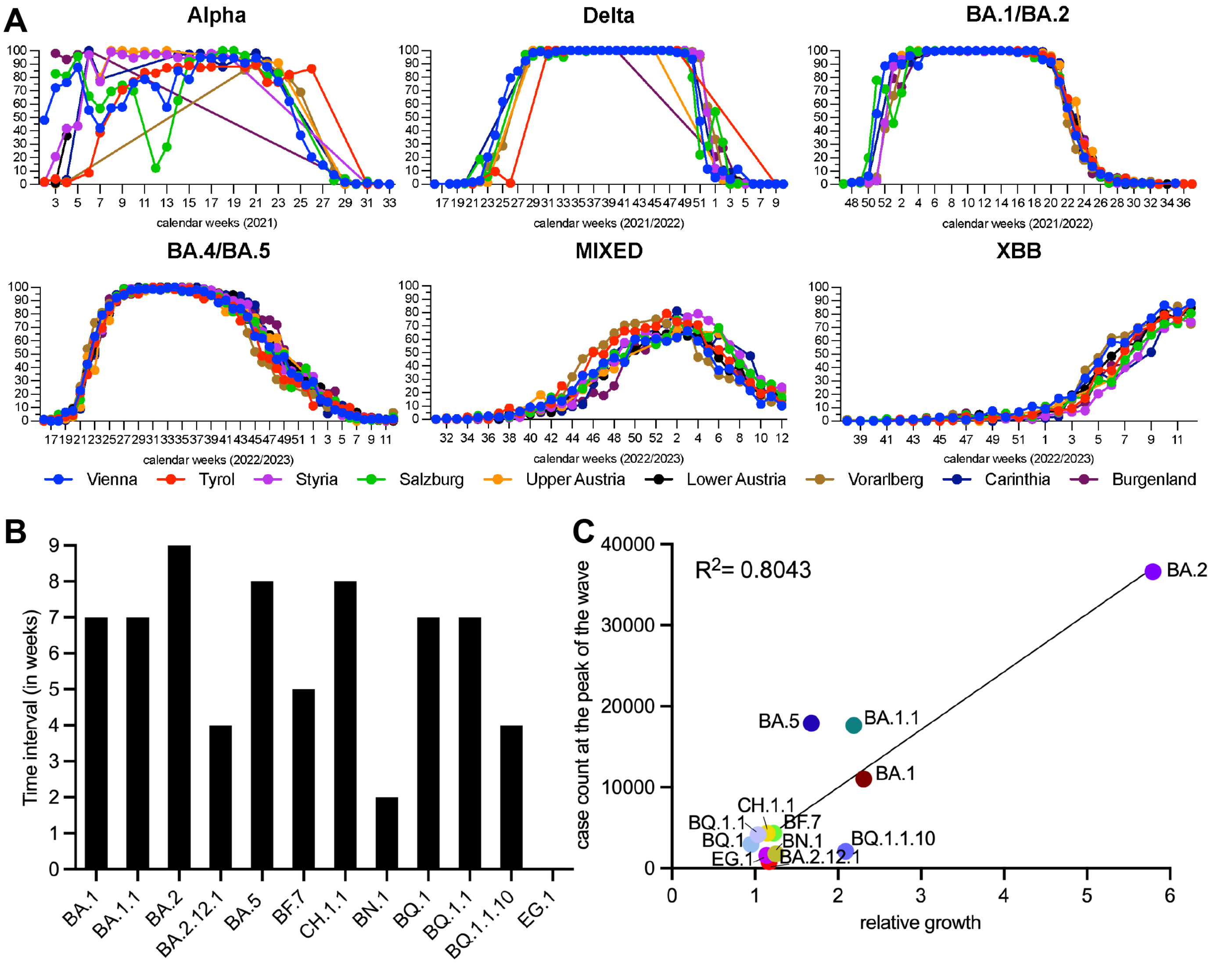

Supplementary Figure S15: Variant dynamics, time to incidence peak, and correlation analysis. A. Fraction of dominant variants per calendar week **B.** Time interval between variants reaching 5% relative share and incidence peak (please refer to **Supplementary Table 4, "variant growth and peak height",** for detailed numerical data). **C.** Correlation between the relative variant growth at the time of reaching a prevalence of around 5% and the case count at the peak of the wave.

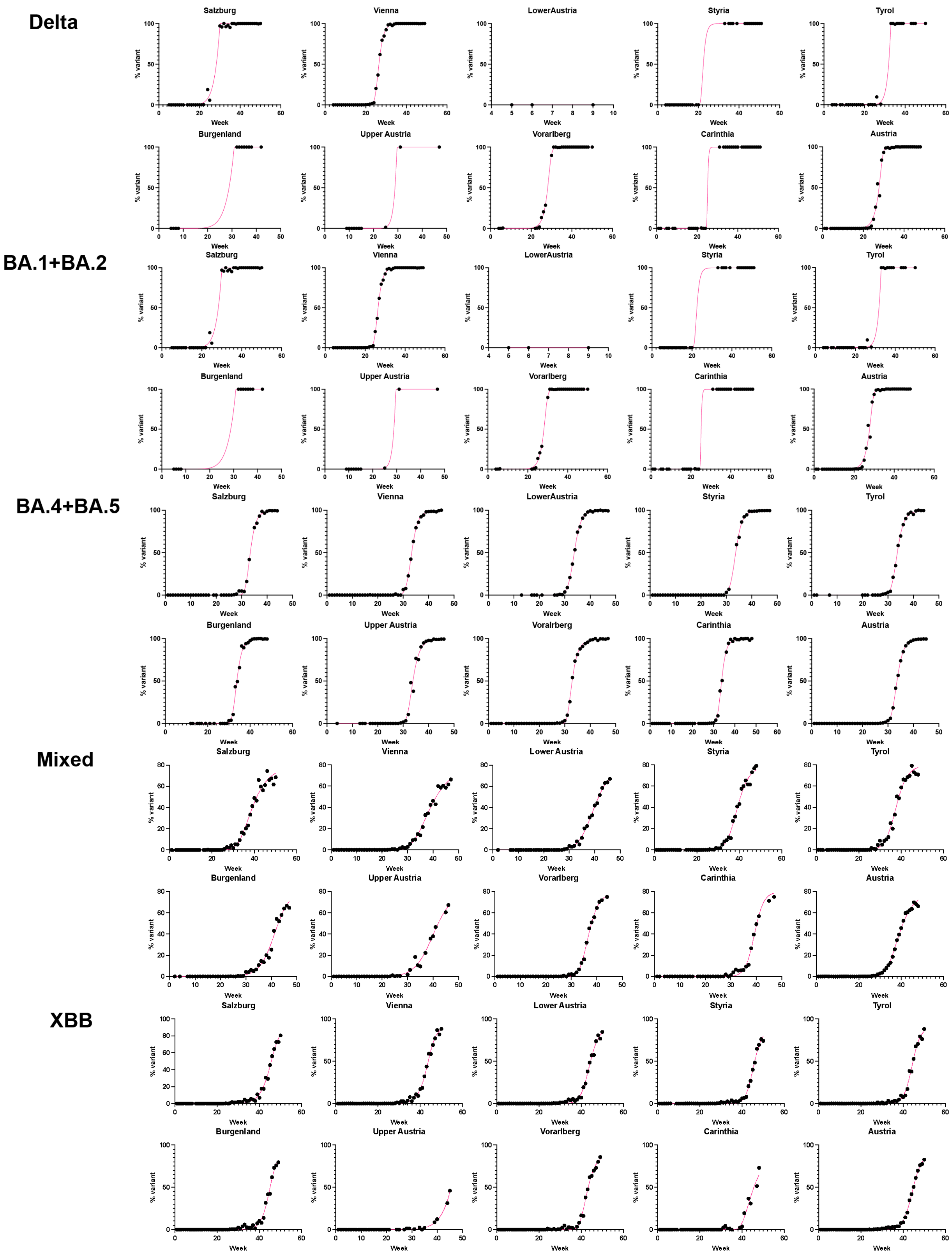

Supplementary Figure S16: Fractions of dominant variants fitted with sigmoidal curves. Fraction of dominant variants plotted per calendar week for each Austrian province and whole Austria. Sigmoidal curves were fitted during the growth phase to determine the time to dominance (TTD) of the variants, assessing the transition time from 10% to 60% prevalence (for **Figure 3C and Supplementary Figure S9C,** please refer to **Supplementary Table 4, "TTD"**, for detailed numerical data), and the time of variant appearance at 10% (for **Figure 3D,** please refer to **Supplementary Table 4," variant appearance",** for detailed numerical data).

References:

1. Quick J. nCoV-2019 sequencing protocol V.1. Protocols.io. 2020;

2. Farr1 B, Rajan1 D, Betteridge1 E, Shirley1 L, Quail1 M, Park1 N, et al. COVID-19 ARTIC v3 Illumina library construction and sequencing protocol V.4. Protocols.io. 2021;

3. Meyers LM, Gutiérrez AH, Boyle CM, Terry F, McGonnigal BG, Salazar A, et al. Highly conserved, non-human-like, and cross-reactive SARS-CoV-2 T cell epitopes for COVID-19 vaccine design and validation. npj Vaccines. 2021;6(1):71.

4. Peng Y, Mentzer AJ, Liu G, Yao X, Yin Z, Dong D, et al. Broad and strong memory CD4+ and CD8+ T cells induced by SARS-CoV-2 in UK convalescent individuals following COVID-19. Nat Immunol. 2020;21(11):1336–45.

5. Mateus J, Grifoni A, Tarke A, Sidney J, Ramirez SI, Dan JM, et al. Selective and cross-reactive SARS-CoV-2 T cell epitopes in unexposed humans. Science. 2020;370(6512):89–94.

6. Yelagandula R, Bykov A, Vogt A, Heinen R, Özkan E, Strobl MM, et al. Multiplexed detection of SARS-CoV-2 and other respiratory infections in high throughput by SARSeq. Nat Commun. 2021;12(1):3132.

7. Sinha R, Stanley G, Gulati GS, Ezran C, Travaglini KJ, Wei E, et al. Index switching causes “spreading-of-signal” among multiplexed samples in Illumina HiSeq 4000 DNA sequencing. bioRxiv. 2017;125724.

8. MacConaill LE, Burns RT, Nag A, Coleman HA, Slevin MK, Giorda K, et al. Unique, dual-indexed sequencing adapters with UMIs effectively eliminate index cross-talk and significantly improve sensitivity of massively parallel sequencing. BMC Genom. 2018;19(1):30.

9. Wölfel R, Corman VM, Guggemos W, Seilmaier M, Zange S, Müller MA, et al. Virological assessment of hospitalized patients with COVID-2019. Nature. 2020;581(7809):465–9.

10. CDC’s Diagnostic Test for COVID-19 Only and Supplies. Available from: https://stacks.cdc.gov/view/cdc/107943

11. Popa A, Genger JW, Nicholson MD, Penz T, Schmid D, Aberle SW, et al. Genomic epidemiology of superspreading events in Austria reveals mutational dynamics and transmission properties of SARS-CoV-2. Sci Transl Med. 2020;12(573):eabe2555.
